# Supplementary material for: Psychological booster shots targeting memory increase long-term resistance against misinformation
Source: Nat Commun. 2025 Mar 11;16:2062. doi: 10.1038/s41467-025-57205-x (PMC11897321; doi:10.1038/s41467-025-57205-x)
Supplement: Supplementary file 1 — Supplementary Information [file 41467_2025_57205_MOESM1_ESM.pdf]

Psychological Booster Shots Targeting Memory Increase Long-Term Resistance Against Misinformation

SUPPLEMENTARY INFORMATION

Rakoen Maertens[1*], Jon Roozenbeek[2,3], Jon S. Simons[2], Stephan Lewandowsky[4,5], Vanessa Maturo[6], Beth Goldberg[6], Rachel Xu[6], and Sander van der Linden[2]

[1] Department of Experimental Psychology, University of Oxford, Oxford, UK

[2] Department of Psychology, University of Cambridge, Cambridge, UK

[3] Department of War Studies, King's College London, London, UK

[4] School of Psychological Science, University of Bristol, Bristol, UK

[5] University of Potsdam, Potsdam, Germany

[6] Jigsaw, Google LLC, New York, NY, USA

* Correspondence concerning this article should be addressed to Rakoen Maertens, Department of Experimental Psychology, University of Oxford, Oxford, UK. Email: rakoen.maertens@psy.ox.ac.uk.

Supplementary Figures **4**
Supplementary Figure 1: (Study 1) SEM of the Memory-Motivation Model at T30 4
Supplementary Figure 2: (Study 1) Inoculation Effects Over Time by Subgroup 5
Supplementary Figure 3: (Study 2) SEM of the Memory-Motivation Model at T0 6
Supplementary Figure 4: (Study 2) Inoculation Effects Over Time by Subgroup 7
Supplementary Figure 5: (Study 3) Plot of Manipulativeness Discernment and Memory 8
Supplementary Figure 6: (Study 4) Plot of Manipulativeness Discernment and Memory 9
Supplementary Figure 7: (Study 4) The Memory-Motivation Model of Inoculation 10
Supplementary Figure 8: (Study 5) Word Cloud of Memory Recall Responses at T30 11
Supplementary Figure 9: (Studies 3–5) Inoculation Effect Across Political Leaning 12
Supplementary Figure 10: (Studies 3–5) Inoculation Effect by Memory and Time 13
Supplementary Figure 11: (Study 3) Inoculation Effects Over Time by Subgroup 14
Supplementary Figure 12: (Study 4) Inoculation Effects Over Time by Subgroup 16
Supplementary Figure 13: (Study 5) Inoculation Effects Over Time by Subgroup 18
Supplementary Figure 14: (Studies 1–5) Inoculation Effects and Memory by Paradigm 20
Supplementary Figure 15: (Study 1) Consensus Message and Misinformation Message 22
Supplementary Figure 16: (Study 1) Flowchart of Experimental Procedure 23
Supplementary Figure 17: (Study 2) Screenshot of the Bad News Game Environment 24
Supplementary Figure 18: (Study 2) Example of Test Item 25
Supplementary Figure 19: (Study 2) Flowchart of Experimental Procedure 26
Supplementary Figure 20: (Study 3) Screenshot of Inoculation Video 27
Supplementary Figure 21: (Studies 3–5) Example of Misleading Social Media Item 28
Supplementary Figure 22: (Study 3) Flowchart of Experimental Procedure 29
Supplementary Figure 23: (Study 4) Flowchart of Experimental Procedure 30
Supplementary Figure 24: (Study 5) Flowchart of Experimental Procedure 31
Supplementary Tables **32**
Supplementary Table 1: (Study 1) Overview of Preregistered Hypotheses 32
Supplementary Table 2: (Study 1) Memory-Motivation Model Estimates 33
Supplementary Table 3: (Study 1) Dominance Analysis at T30 34
Supplementary Table 4: (Study 2) Overview of Preregistered Hypotheses 35
Supplementary Table 5: (Study 2) Memory-Motivation Model Estimates 36
Supplementary Table 6: (Study 2) Dominance Analysis at T30 37
Supplementary Table 7: (Studies 3–5) Overview of Preregistered Hypotheses 38
Supplementary Table 8: (Study 4) Memory-Motivation Model Estimates 40
Supplementary Table 9: (Study 5) Memory-Motivation Model Estimates 41
Supplementary Table 10: (Study 5) Dominance Analysis at T30 42
Supplementary Methods **43**
(Study 1) Information About the Climate Change Inoculation Paradigm 43
(Study 2) Information About the Bad News Inoculation Paradigm 45
(Studies 3–5) Information About the Video-Based Inoculation Paradigm 47

Supplementary Discussion **49**
Mapping and Boosting the Longevity of Counter-Misinformation Interventions 49
Integrating Memory Research and Inoculation Theory 54
Conceptual and Methodological Issues in Longitudinal Inoculation 56
Supplementary Notes **59**
Supplementary Note 1: (Study 1) SEM Model Estimates Analysis 59
Supplementary Note 2: (Study 1) Underlying Mechanisms Dominance Analysis 60
Supplementary Note 3: (Study 2) SEM Model Estimates Analysis 61
Supplementary Note 4: (Study 2) Underlying Mechanisms Dominance Analysis 62
Supplementary Note 5: (Study 3) Evidence for Hypotheses Analysis 63
Supplementary Note 6: (Study 4) Evidence for Hypotheses Analysis 65
Supplementary Note 7: (Study 5) SEM Model Estimates Analysis 67
Supplementary Note 8: (Study 5) Underlying Mechanisms Dominance Analysis 69
Supplementary Note 9: (Study 5) Open Memory Questions Word Cloud Analysis 70
References **71**

Supplementary Figures

Supplementary Figure 1: *(Study 1) SEM of the Memory-Motivation Model at T30*

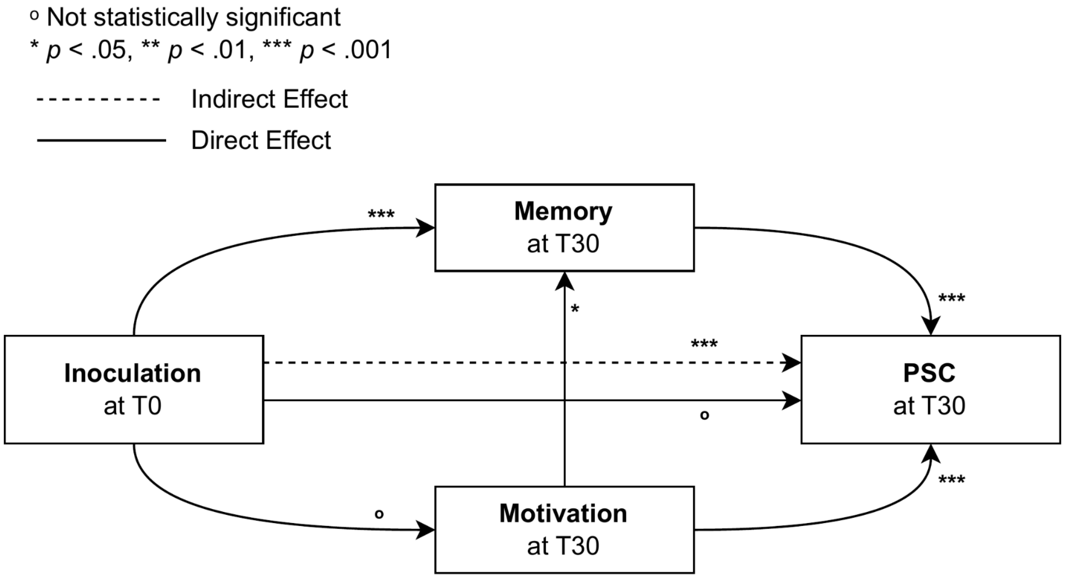

This figure represents the direct and indirect effects of inoculation on the perceived scientific consensus (PSC) ~30 days later (T30), through objective memory recall (Memory) and motivation to defend oneself against misinformation (Motivation). *N* = 689.

Supplementary Figure 2: *(Study 1) Inoculation Effects Over Time by Subgroup*

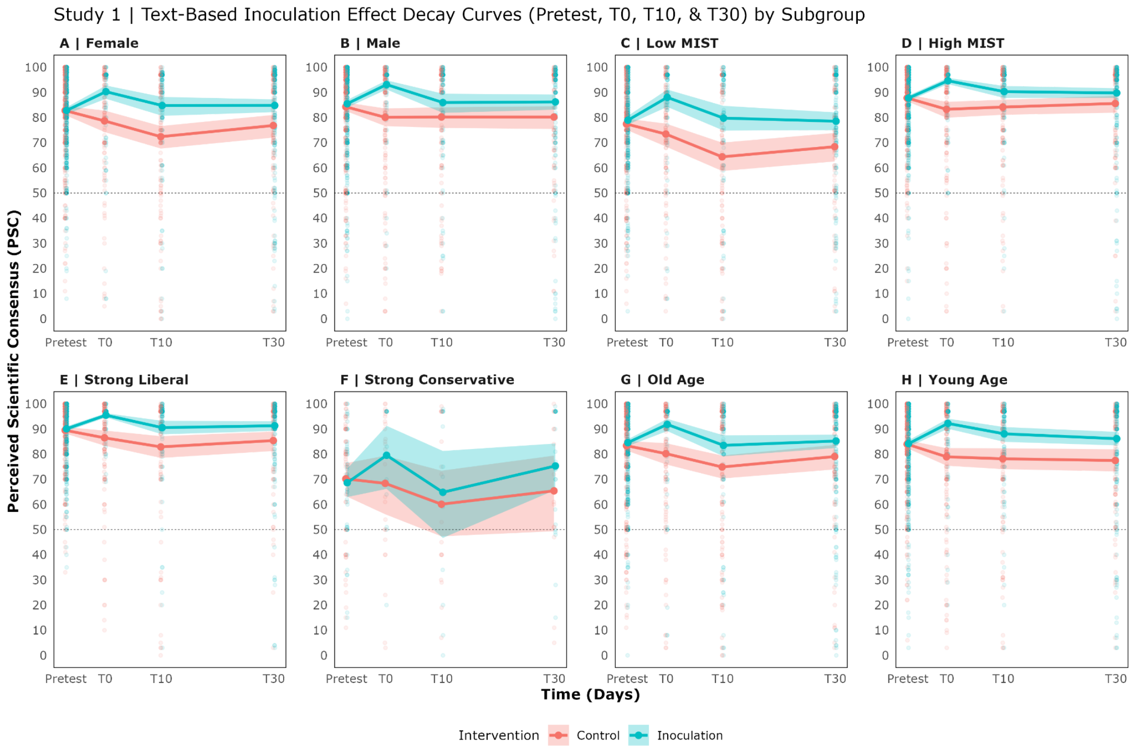

Line graphs of the inoculation effects over time for participant subgroups. All panels represent the text-based inoculation effect in Study 1. Panel A–B: inoculation effect in gender subgroups (n_{female} = 880, n_{male} = 898). Panel C–D: inoculation effect in median split misinformation susceptibility subgroups (n_{lowMIST} = 714, n_{highMIST} = 1,111). Panel E–F: inoculation effect in political ideology subgroups ($n_{\text{strongLiberal}}$ = 855, $n_{\text{strongConservative}}$ = 109). Panel G–H: inoculation effect in median split age subgroups (n_{oldAge} = 879, n_{youngAge} = 946). Control (red line) = control group that received no inoculation intervention. Inoculation (green line) = inoculation group that received an inoculation intervention. Error bands represent 95% confidence intervals around the mean.

Supplementary Figure 3: *(Study 2) SEM of the Memory-Motivation Model at T0*

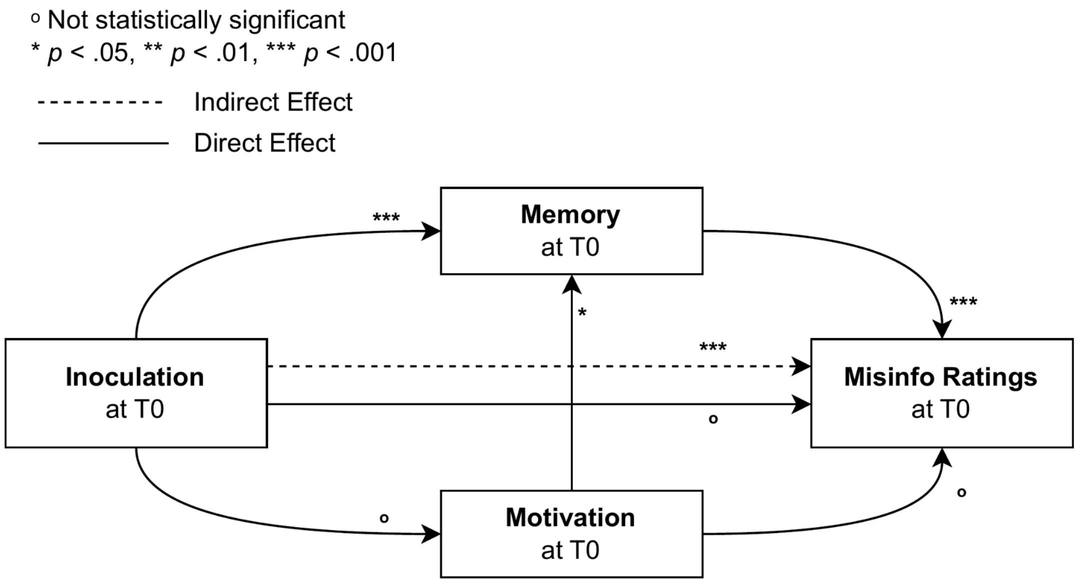

This figure represents the direct and indirect effects of inoculation on the reliability ratings of misinformation items (Misinfo Ratings) immediately after the intervention (T0), through objective memory recall (Memory) and motivation to defend oneself against misinformation (Motivation). $N = 319$.

Supplementary Figure 4: *(Study 2) Inoculation Effects Over Time by Subgroup*

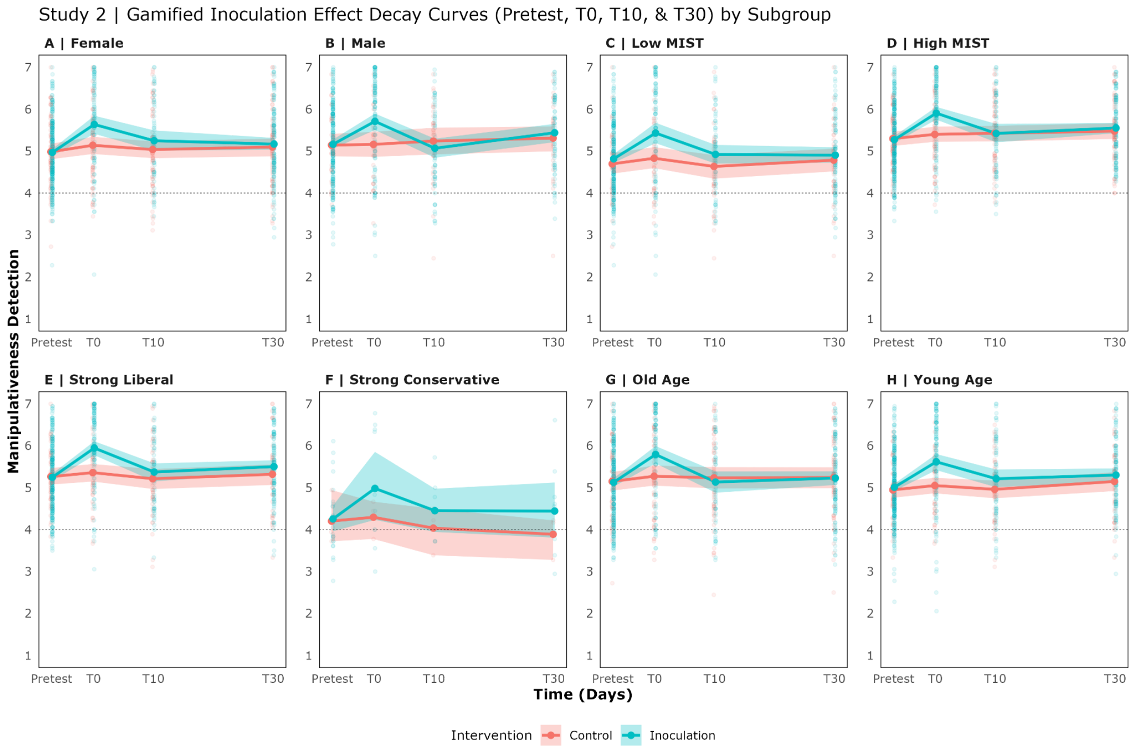

Line graphs of the inoculation effects over time for participant subgroups. All panels represent the gamified inoculation effect in Study 2. Panel A–B: inoculation effect in gender subgroups ($n_{\text{female}} = 436$, $n_{\text{male}} = 311$). Panel C–D: inoculation effect in median split misinformation susceptibility subgroups ($n_{\text{lowMIST}} = 342$, $n_{\text{highMIST}} = 440$). Panel E–F: inoculation effect in political ideology subgroups ($n_{\text{strongLiberal}} = 368$, $n_{\text{strongConservative}} = 32$). Panel G–H: inoculation effect in median split age subgroups ($n_{\text{oldAge}} = 386$, $n_{\text{youngAge}} = 396$). Control (red line) = control group that received no inoculation intervention. Inoculation (green line) = inoculation group that received an inoculation intervention. Error bands represent 95% confidence intervals around the mean.

Supplementary Figure 5: *(Study 3) Plot of Manipulativeness Discernment and Memory*

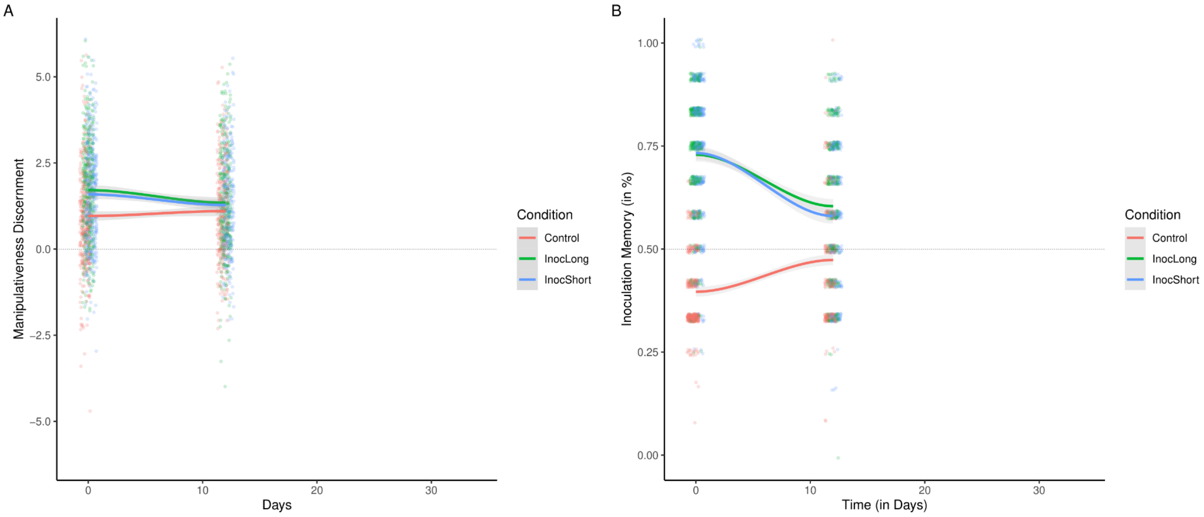

The figure shows the manipulativeness discernment (Panel A) and inoculation memory (Panel B) over time (in days) across three groups: Control (red line), InocLong (green line; long inoculation video of 1 minute 48 seconds), and InocShort (blue line; short inoculation video of 30 seconds). Error bands represent 95% confidence intervals around the mean. The sample size for the study is $N = 2{,}219$.

Supplementary Figure 6: *(Study 4) Plot of Manipulativeness Discernment and Memory*

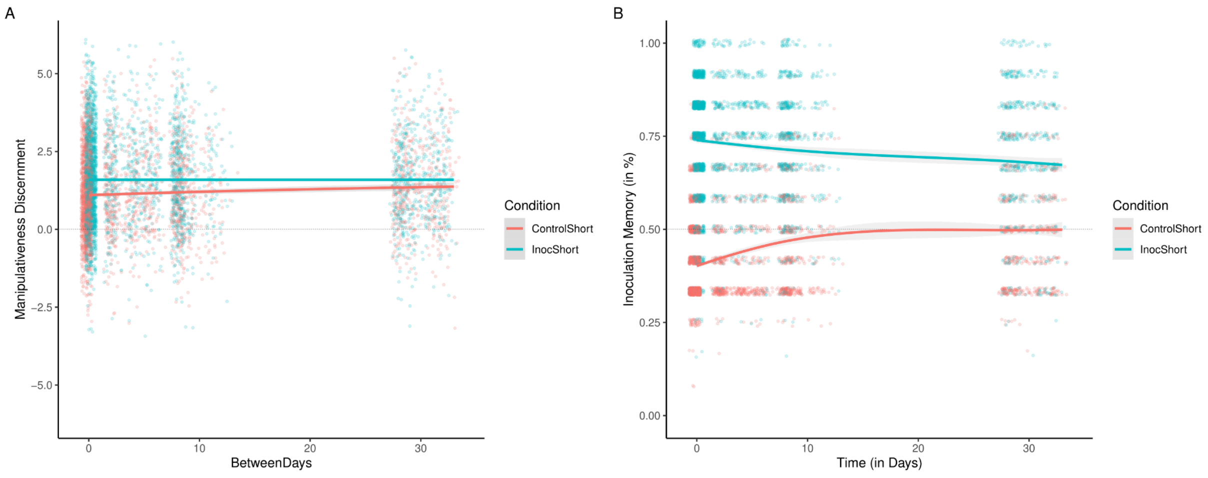

The figure shows changes in manipulativeness discernment (Panel A) and inoculation memory (Panel B) over time (in days) across two groups: ControlShort (red line; 30-second control video) and InocShort (blue line; 30-second inoculation video). Days represent the time elapsed since the inoculation intervention. Error bands represent 95% confidence intervals around the mean. The sample size for the study is $N = 3{,}066$.

Supplementary Figure 7: *(Study 4) The Memory-Motivation Model of Inoculation*

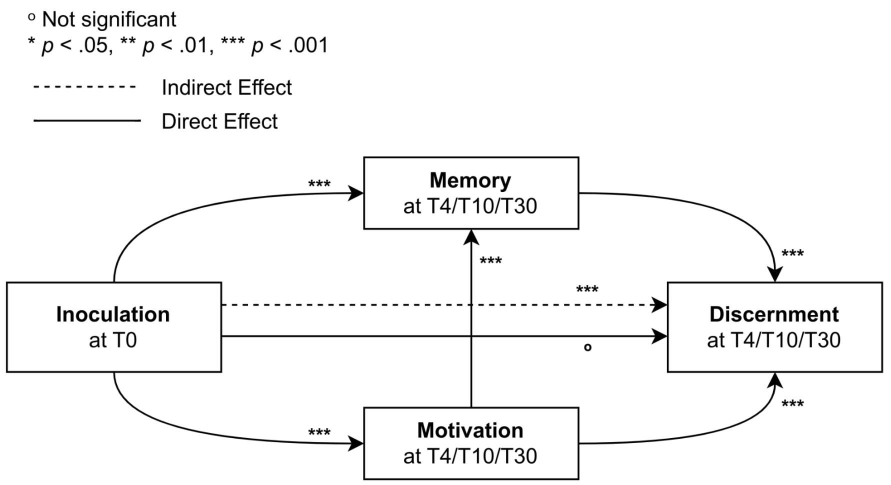

This figure represents the direct and indirect effects of inoculation on the discernment of manipulative items from neutral items (Discernment) at a later date, through objective memory recall (Memory) and motivation to defend oneself against misinformation (Motivation). $N = 3{,}066$.

Supplementary Figure 8: *(Study 5) Word Cloud of Memory Recall Responses at T30*

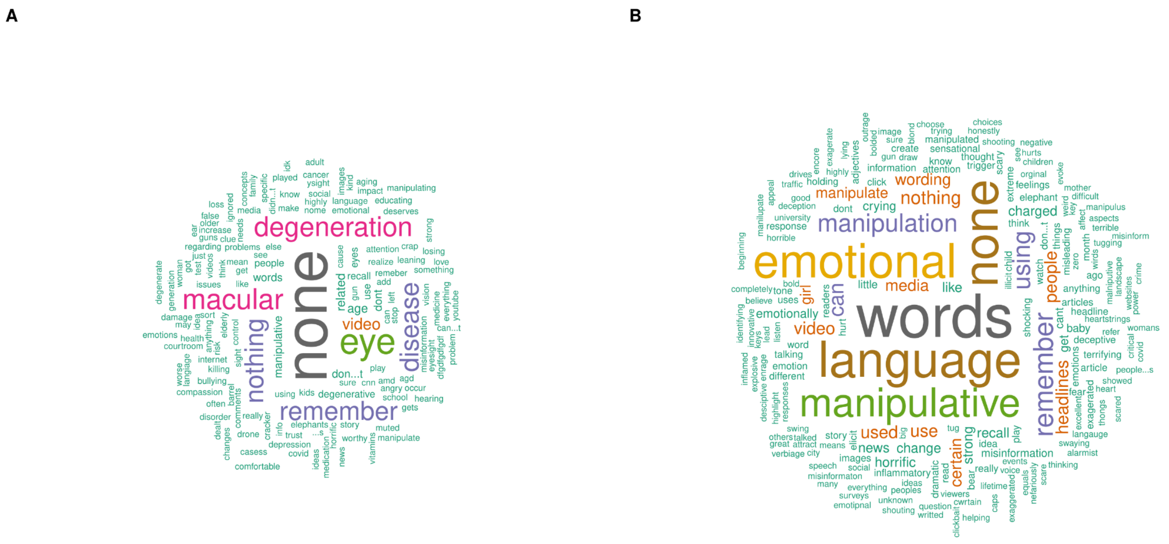

Word Cloud of memory recall question responses at T30 for participants in the control group (Panel A) and in the inoculation group that received a memory booster (Panel B) in Study 5. Larger words represent a higher occurrence of the word. The visualisations were generated using the R packages wordcloud (2.6) [1] and RColorBrewer (1.1.3) [2]. $N = 626$.

Supplementary Figure 9: *(Studies 3–5) Inoculation Effect Across Political Leaning*

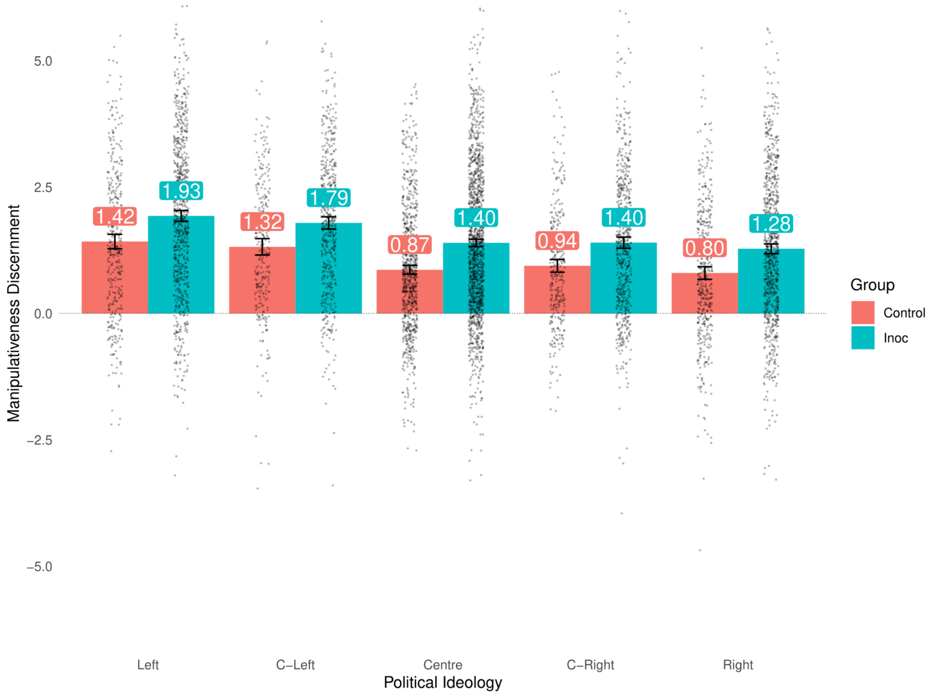

The figure shows differences in manipulativeness discernment across political ideologies, comparing the Control group (red bars) and the Inoculation group (blue bars) in the combined sample of Studies 3–5. Political ideology is categorized from Left to Right along the x-axis, with manipulativeness discernment plotted on the y-axis. Error bars represent 95% confidence intervals around the mean. The sample size for the analysis is $N_{\text{datapoints}}$ = 6,518.

Supplementary Figure 10: *(Studies 3–5) Inoculation Effect by Memory and Time*

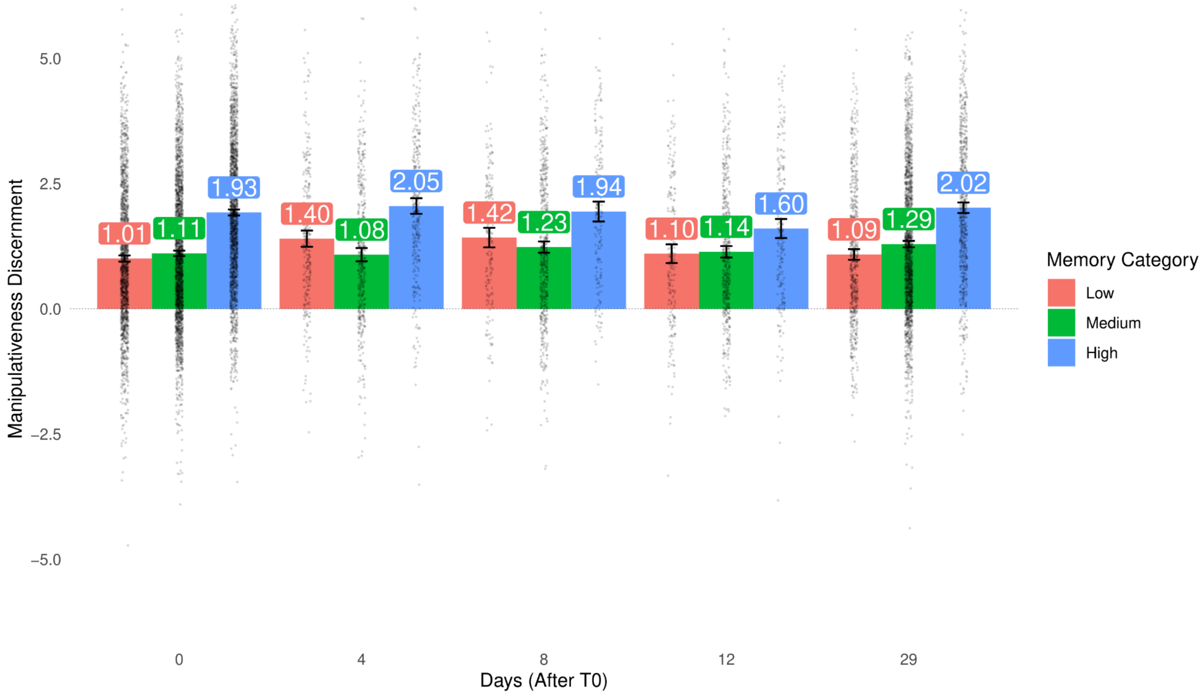

The figure shows changes in manipulativeness discernment over time (in days) after the intervention, categorized by inoculation memory recall levels: Low (red bars), Medium (green bars), and High (blue bars). Days after the intervention (T0) are indicated on the x-axis, with time points at ~0, ~4, ~8, ~12, and ~29 days. Error bars represent 95% confidence intervals around the mean. The sample size for the study is $N_{\text{datapoints}}$ =12,791.

Supplementary Figure 11: *(Study 3) Inoculation Effects Over Time by Subgroup*

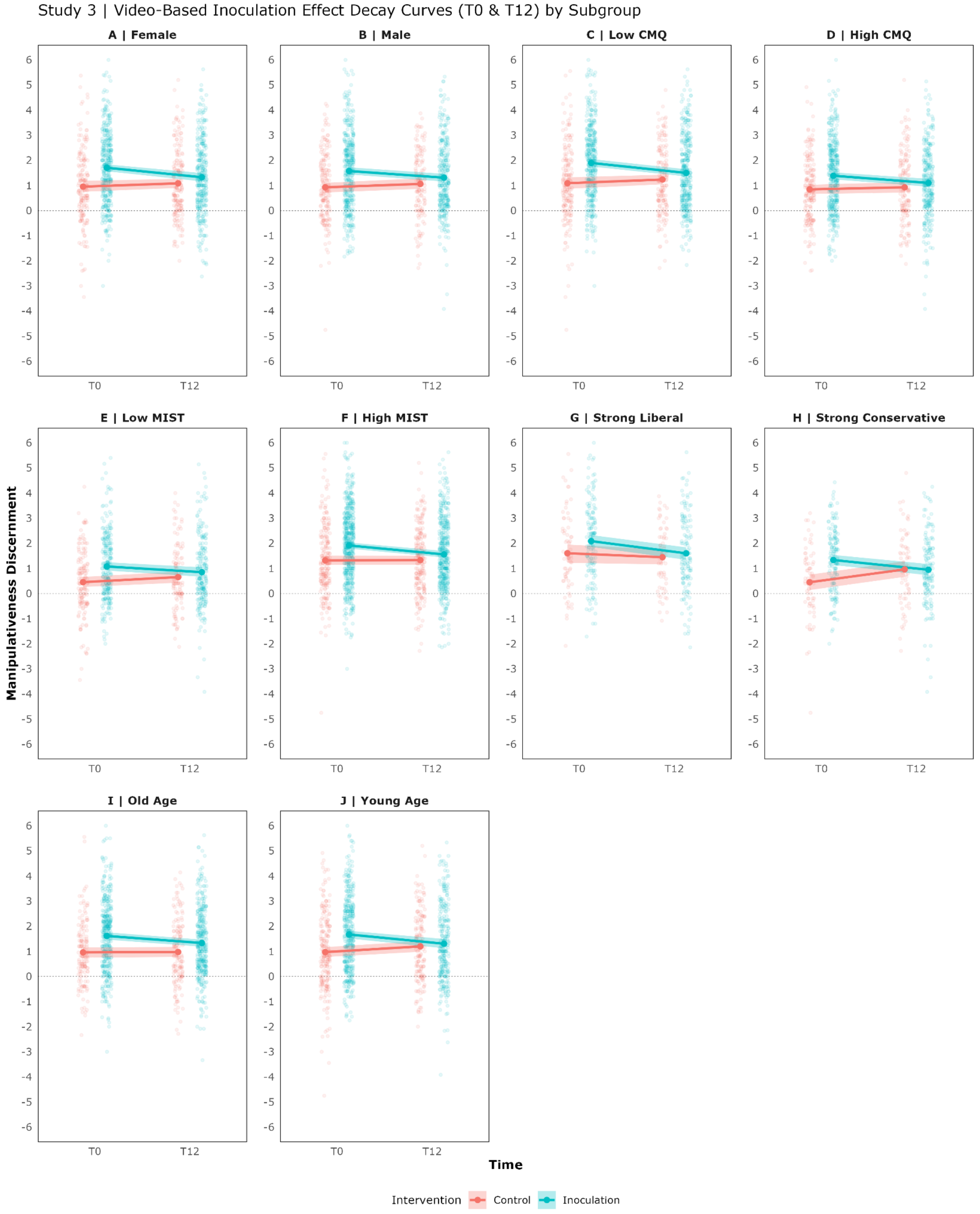

Line graphs of the inoculation effects over time for participant subgroups. All panels represent the video-based inoculation effect in Study 3. Panel A–B: inoculation effect in gender subgroups (n_{female} = 1,066, n_{male} = 1,048). Panel C–D: inoculation effect in median split conspiracy mentality subgroups (n_{lowCMQ} = 1,099, n_{highCMQ} = 1,038). Panel E–F: inoculation

effect in median split misinformation susceptibility subgroups ($n_{lowMIST}$ = 744, $n_{highMIST}$ = 1,393). Panel G–H: inoculation effect in political ideology subgroups ($n_{strongLiberal}$ = 425, $n_{strongConservative}$ = 435). Panel I–J: inoculation effect in median split age subgroups (n_{oldAge} = 1,067, $n_{youngAge}$ = 1,070). Control (red line) = control group that received no inoculation intervention. Inoculation (green line) = inoculation group that received an inoculation intervention. Error bands represent 95% confidence intervals around the mean.

Supplementary Figure 12: *(Study 4) Inoculation Effects Over Time by Subgroup*

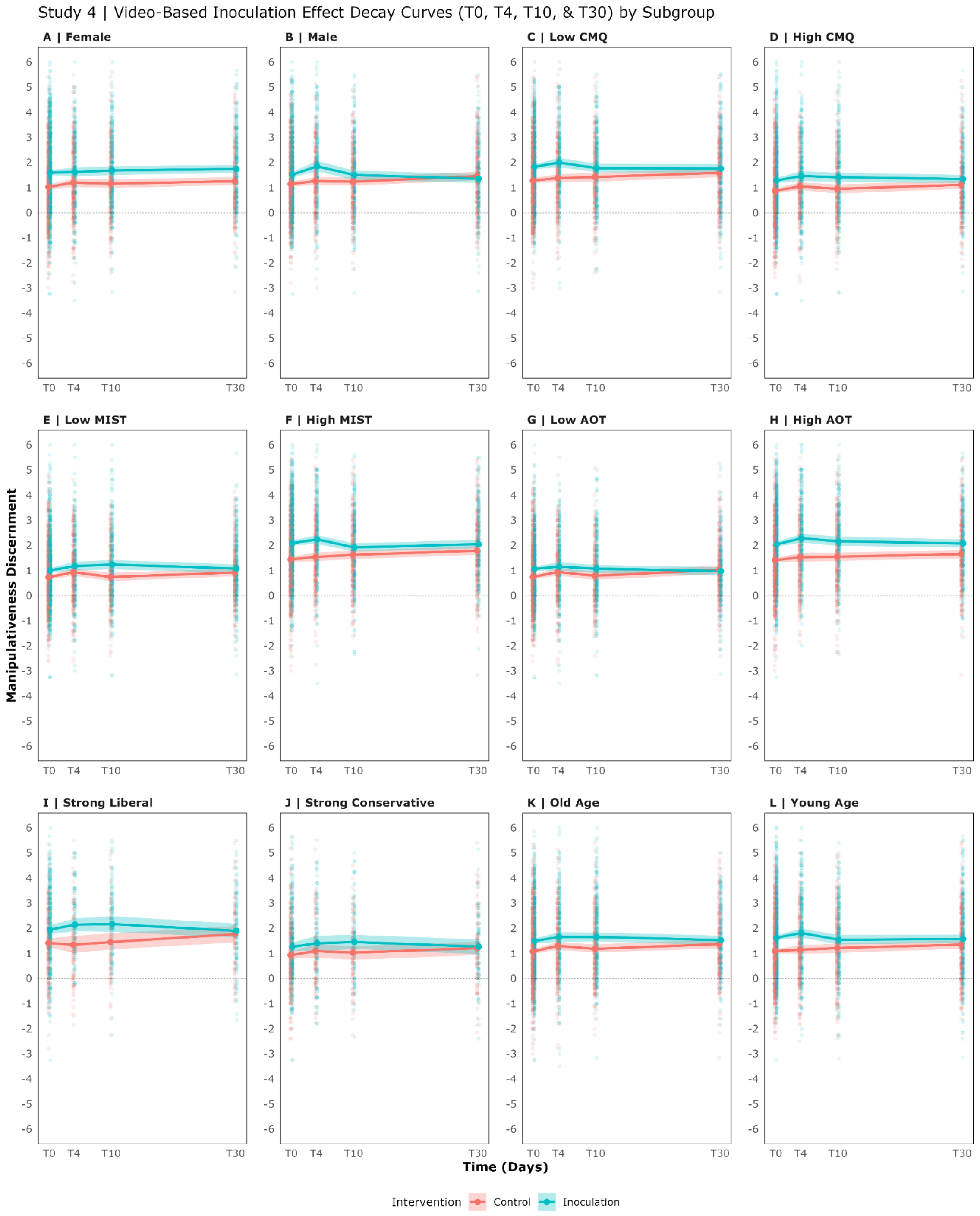

Line graphs of the inoculation effects over time for participant subgroups. All panels represent the video-based inoculation effect in Study 4. Panel A–B: inoculation effect in gender subgroups (n_{female} = 1,552, n_{male} = 1,435). Panel C–D: inoculation effect in median split conspiracy mentality subgroups (n_{lowCMQ} = 1,539, n_{highCMQ} = 1,459). Panel E–F: inoculation

effect in median split misinformation susceptibility subgroups (n_{lowMIST} = 1,482, n_{highMIST} = 1,516). Panel G–H: inoculation effect in median split actively open-minded thinking subgroups (n_{lowAOT} = 1,472, n_{highAOT} = 1,526). Panel I–J: inoculation effect in political ideology subgroups ($n_{\text{strongLiberal}}$ = 549, $n_{\text{strongConservative}}$ = 592). Panel K–L: inoculation effect in median split age subgroups (n_{oldAge} = 1,450, n_{youngAge} = 1,548). Control (red line) = control group that received no inoculation intervention. Inoculation (green line) = inoculation group that received an inoculation intervention. Error bands represent 95% confidence intervals around the mean.

Supplementary Figure 13: *(Study 5) Inoculation Effects Over Time by Subgroup*

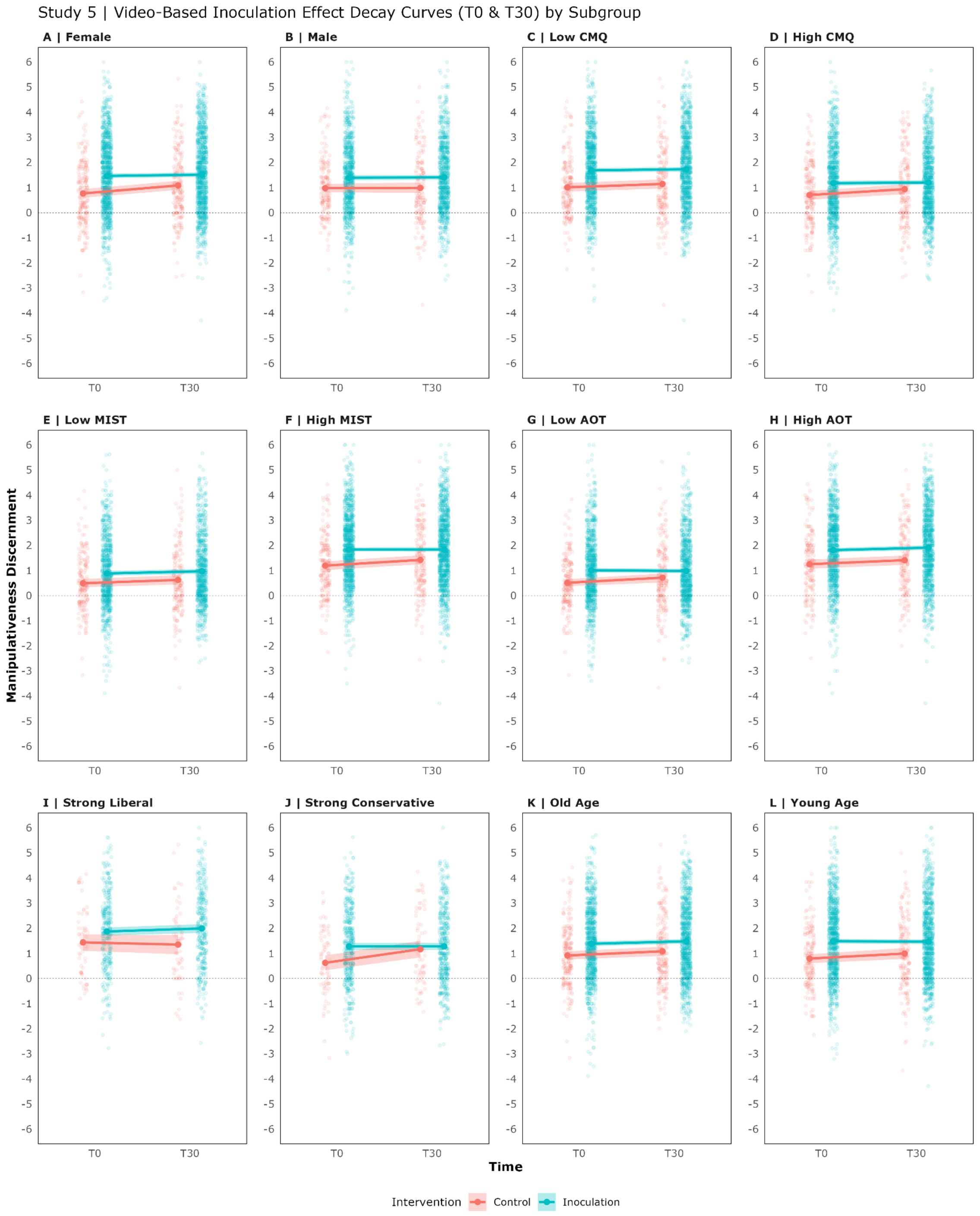

Line graphs of the inoculation effects over time for participant subgroups. All panels represent the video-based inoculation effect in Study 5. Panel A–B: inoculation effect in gender subgroups (n_{female} = 1,224, n_{male} = 988). Panel C–D: inoculation effect in median split conspiracy mentality subgroups (n_{lowCMQ} = 1,122, n_{highCMQ} = 1,098). Panel E–F: inoculation

effect in median split misinformation susceptibility subgroups (n_{lowMIST} = 953, n_{highMIST} = 1,267). Panel G–H: inoculation effect in median split actively open-minded thinking subgroups (n_{lowAOT} = 1,065, n_{highAOT} = 1,155). Panel I–J: inoculation effect in political ideology subgroups ($n_{\text{strongLiberal}}$ = 379, $n_{\text{strongConservative}}$ = 493). Panel K–L: inoculation effect in median split age subgroups (n_{oldAge} = 1,092, n_{youngAge} = 1,128). Control (red line) = control group that received no inoculation intervention. Inoculation (green line) = inoculation group that received an inoculation intervention. Error bands represent 95% confidence intervals around the mean.

Supplementary Figure 14: *(Studies 1–5) Inoculation Effects and Memory by Paradigm*

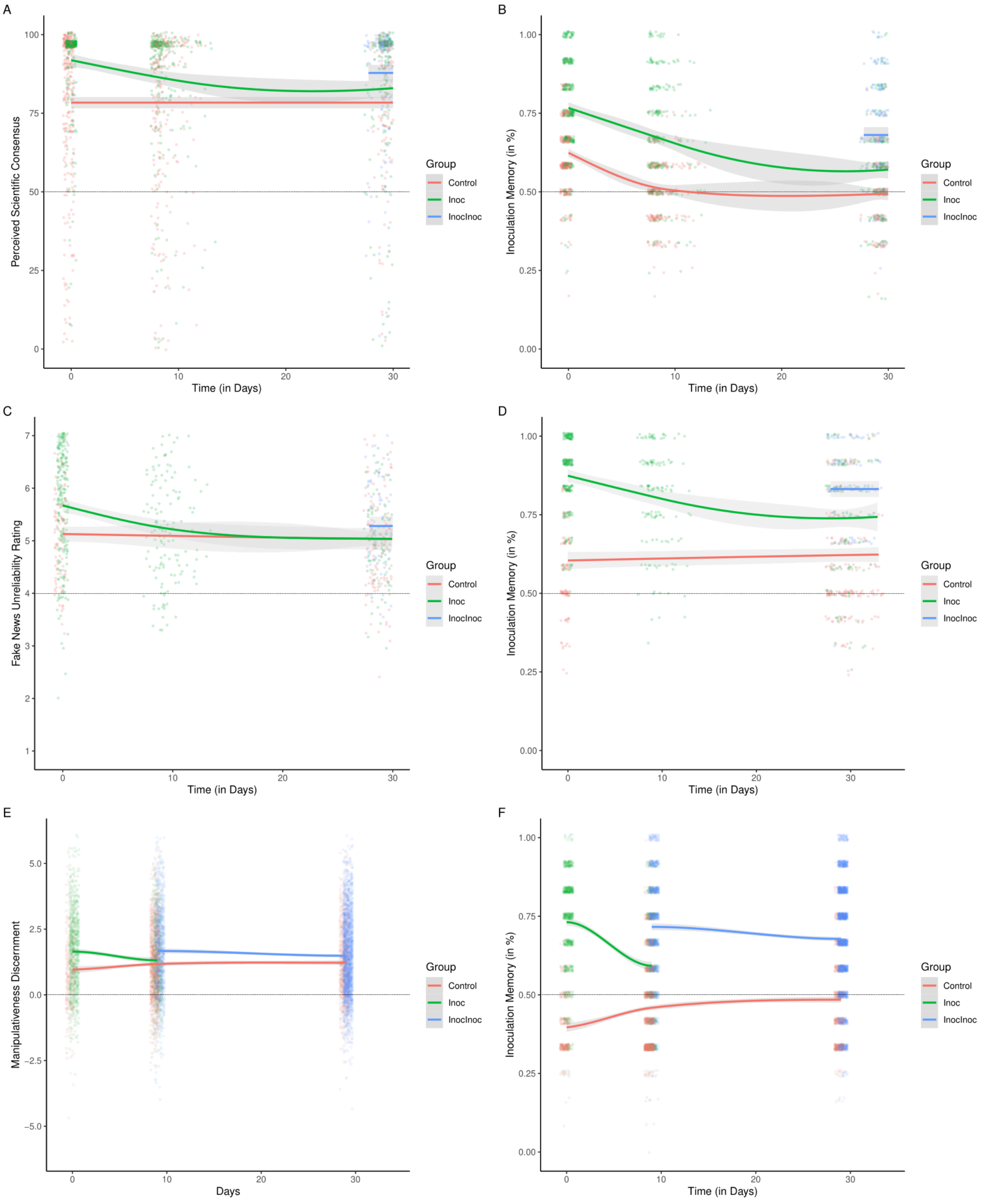

Line graphs of the inoculation effects (first column) and the objective inoculation memory (second column) for each of the three intervention paradigms (Studies 1–5). Panel A–B (Study 1): text-based inoculation ($N_{datapoints}$ = 5,475). Panel C–D (Study 2): gamified inoculation ($N_{datapoints}$ = 2,022). Panel E–F (Studies 3–5): video-based inoculation ($N_{datapoints}$ = 7,505). Control (red line) = control group. Inoc (green line) = single inoculation group.

InocInoc (blue line) = boosted inoculation group. Error bands represent 95% confidence intervals around the mean.

Supplementary Figure 15: *(Study 1) Consensus Message and Misinformation Message*

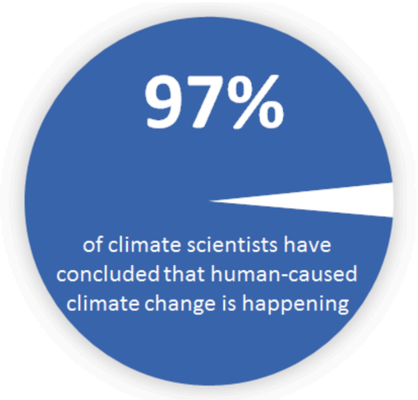

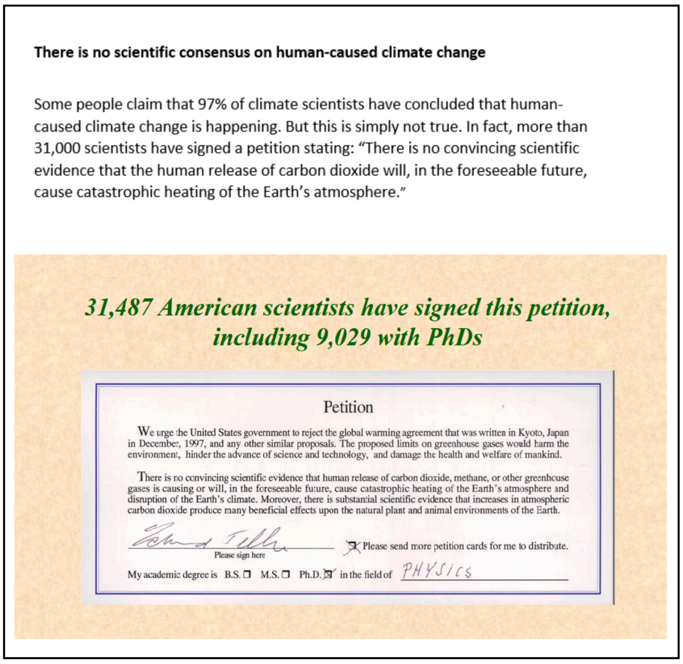

Consensus message (left) and misinformation message (right) used in Study 1. These materials have been adopted (entirely and unmodified) from van der Linden et al. [3] and Maertens et al. [4]. The consensus message is a visualization of the real consensus among climate scientists [5] about anthropogenic global warming and used as a real news stimulus in Study 1 to test the relative impact of the consensus message on the perceived scientific consensus compared to the misinformation message, to help establish the inoculation effects of the intervention. The reason for it being depicted as a pie chart is that it was found by van der Linden et al. [6] to be one of the most effective ways to communicate this scientific consensus. The message on the right contains a screenshot of the Oregon Petition [7], a real but misleading petition in the public domain. It is used in Study 1 as the misinformation message, as it is a petition that contains false signatures and people without any relevant expertise. For a more detailed discussion about these stimuli, see van der Linden et al. [3].

Supplementary Figure 16: *(Study 1) Flowchart of Experimental Procedure*

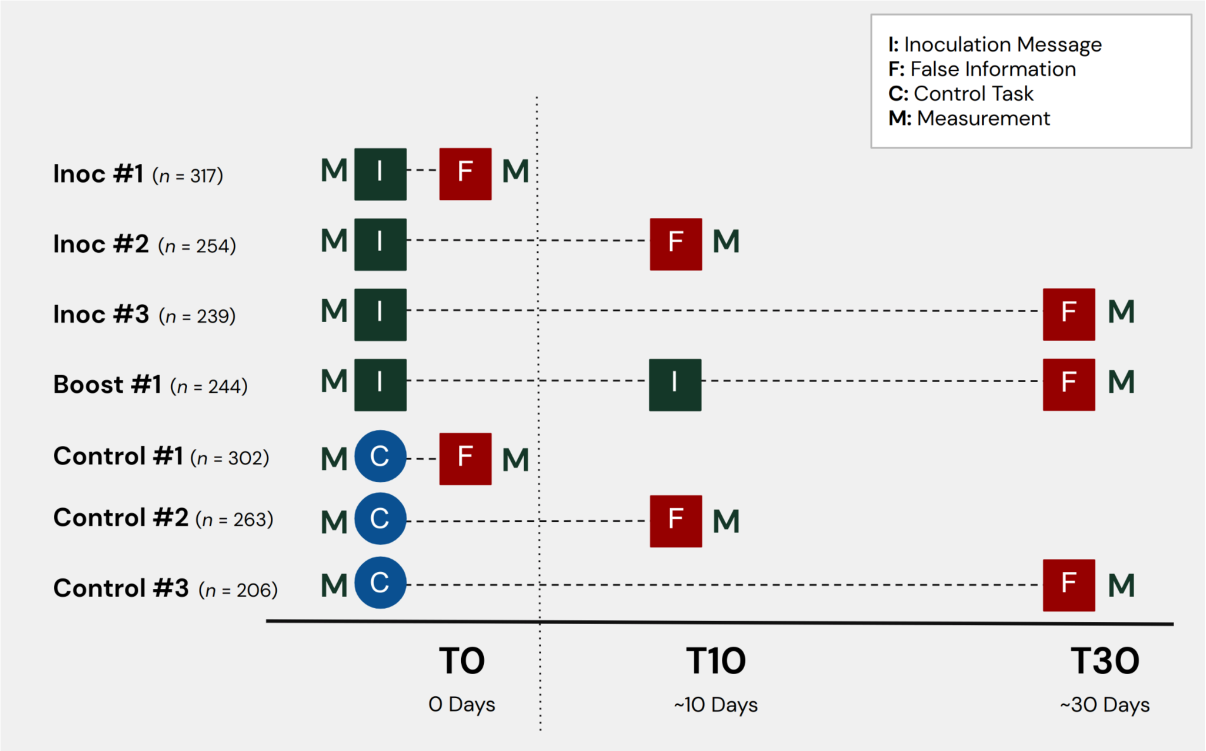

This figure depicts the flow of the experiment for each of the four experimental groups and for the three control groups in Study 1.

Supplementary Figure 17: *(Study 2) Screenshot of the Bad News Game Environment*

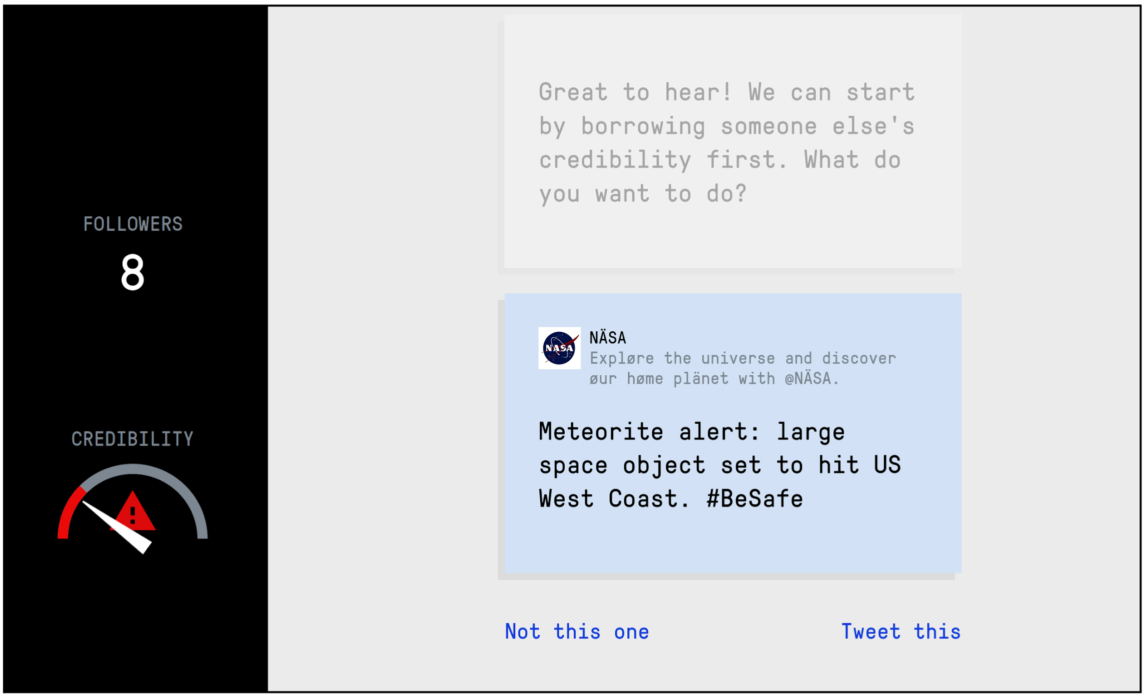

This is a screenshot from the public Bad News inoculation game that was published by Roozenbeek and van der Linden [8] and which has been used (entirely and unmodified) as the main intervention in Study 2. In the game, participants step into the shoes of the misinformation creator as a method to teach people how the disinformer thinks and how misinformation can be easily propagated, thereby protecting (inoculating) the participant. In the game participants have to find a balance between gaining followers (e.g., by posting outrageous content) and keeping their credibility levels high enough. The example social media post depicted here in the screenshot is artificially created and demonstrates the impersonation disinformation technique, which helps participants to understand how fake accounts can mislead people, in this case a fake account using the NASA logo but using the incorrect profile name NÄSA (i.e., with an umlaut on the first A, while NASA does not have any umlauts in their name).

Supplementary Figure 18: *(Study 2) Example of Test Item*

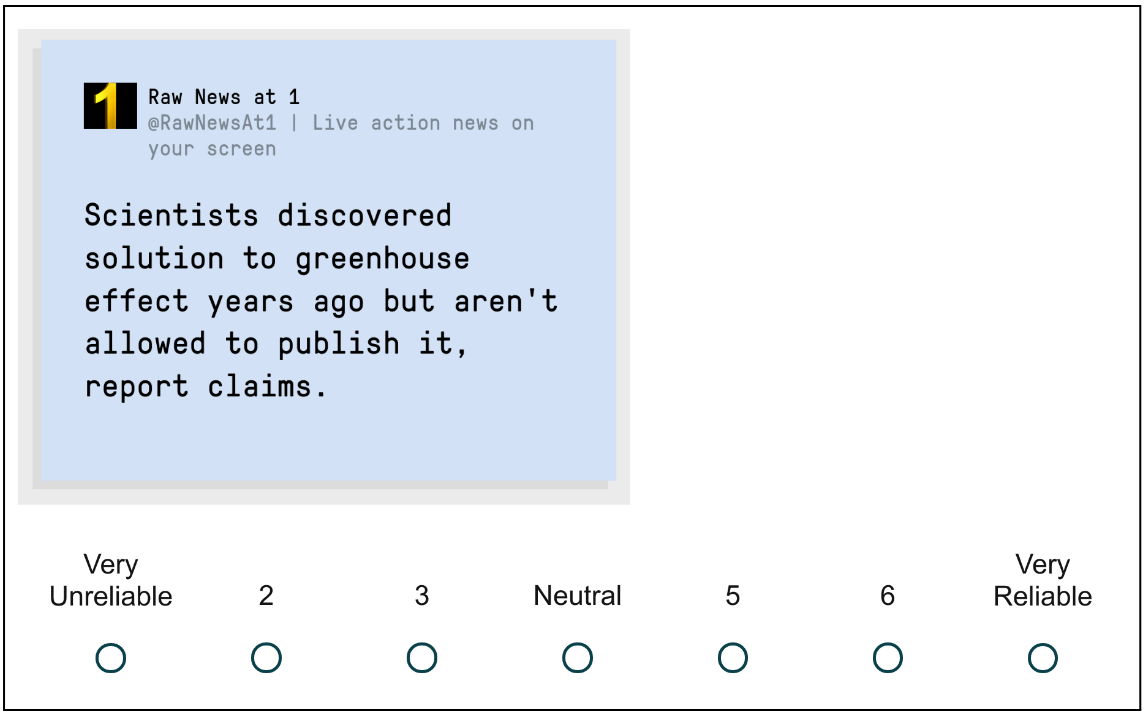

This figure depicts a test item used in Study 2 that makes use of the conspiracy technique. All test items are artificially created and were adopted (the entire item set, unmodified) from Maertens et al. [9]. It resembles a social media type post that could be found on platforms such as X, but in this case is entirely fictional. Participants who have completed the inoculation game Bad News learn about these types of social media posts and what is potentially unreliable about them. In this case, it would be expected that the a conspiracy narrative where for a long time a group of people has known something but is not allowed to tell the truth because of an unknown group, without any explanation of who that group would be or what the evidence is, should make inoculated people more sceptical as they recognize the narrative (i.e., rate this post as less reliable on the presented reliability scale from 1–7). This item and similar items were used as pretest and posttest in Study 2 to measure the inoculation effect.

Supplementary Figure 19: *(Study 2) Flowchart of Experimental Procedure*

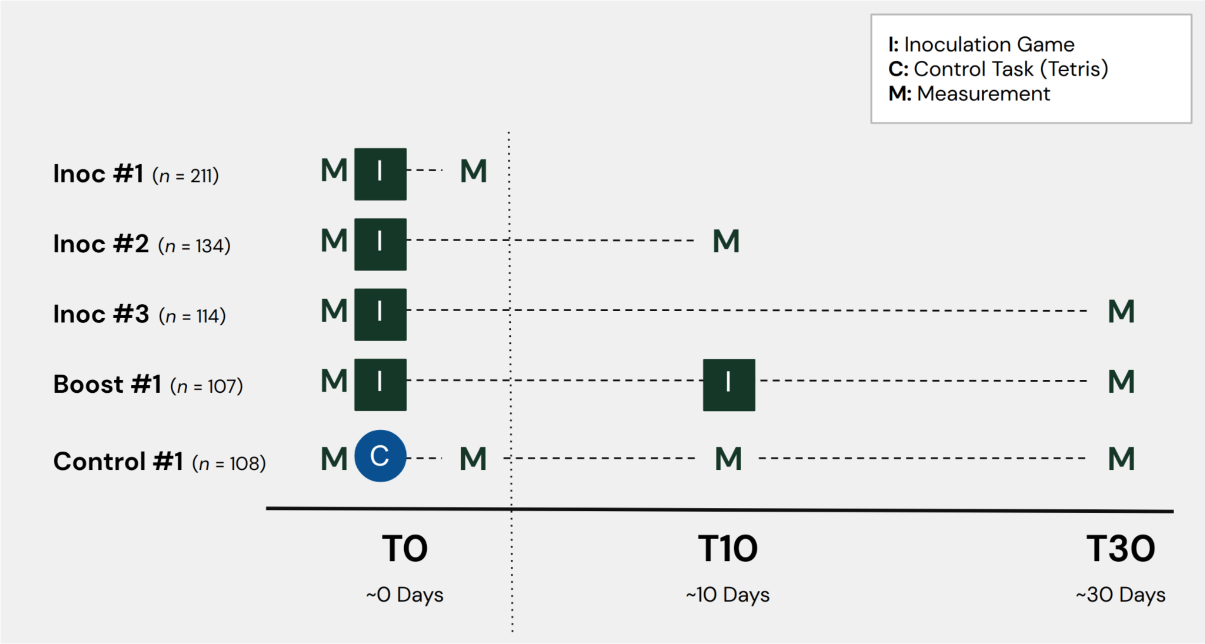

This figure depicts the flow of the experiment for each of the four experimental groups and for the control group in Study 2.

Supplementary Figure 20: *(Study 3) Screenshot of Inoculation Video*

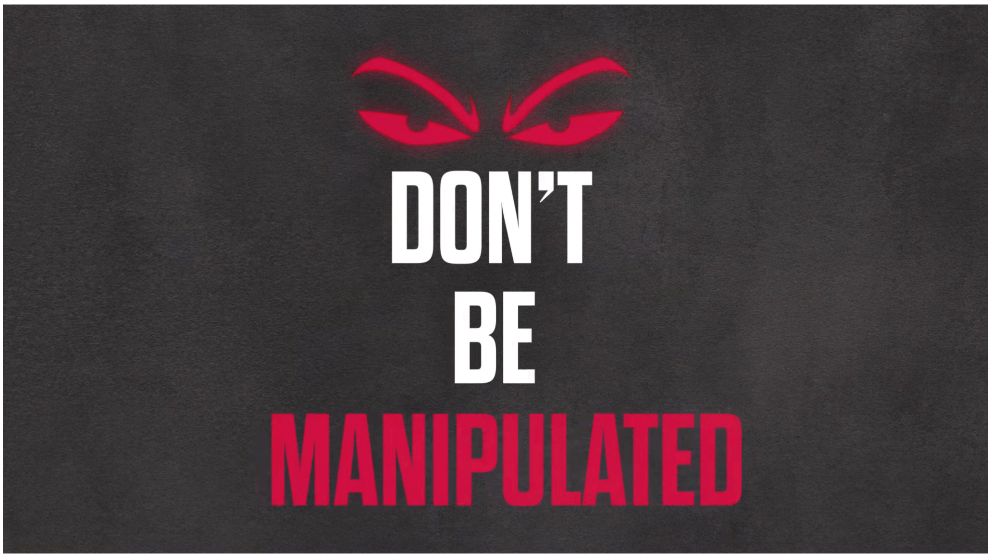

This is a screenshot of the video-based emotional language inoculation intervention that was published by Roozenbeek et al. [10] and which has been used (entirely and unmodified) as the long video-based inoculation intervention in Study 3. The video features examples of how people can be fooled by the excessive use of emotional language to manipulate people in a certain direction. It ends with the affective forewarning message that can be seen in this screenshot, which aims to motivate participants to resist misinformation by boosting perceived threat levels. This video, as well as other inoculation videos, can be found on https://inoculation.science/inoculation-videos/.

Supplementary Figure 21: *(Studies 3–5) Example of Misleading Social Media Item*

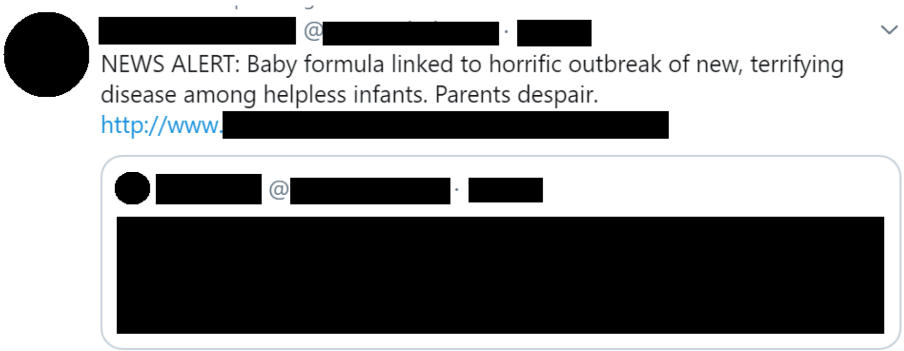

This figure depicts a test item used in Studies 3–5 that makes use of the emotional language manipulation technique. All test items are artificially created and were adopted (the entire item set, unmodified) from Roozenbeek et al. [10]. It resembles a social media type post that could be found on platforms such as X, but in this case is entirely fictional. Participants who have watched the inoculation video learn about how content that uses emotional language can manipulate people. In this case, it is expected that the use of strongly emotional language such as “horrific”, “terrifying”, “helpless”, and “despair” in a single post, would make inoculated people more sceptical as they recognize the emotional narrative (i.e., they would rate this post as more manipulative). This item and similar items were used for the posttest measure of the inoculation effect in Studies 3–5.

Supplementary Figure 22: *(Study 3) Flowchart of Experimental Procedure*

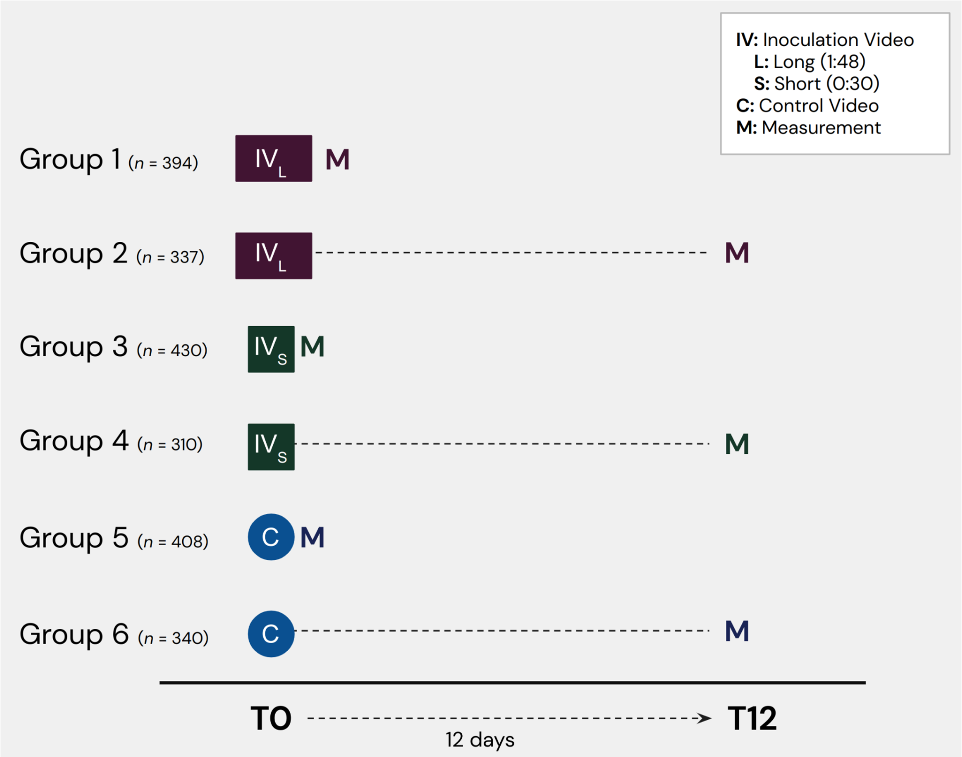

This figure depicts the flow of the experiment for each of the four experimental groups and for the two control groups in Study 3.

Supplementary Figure 23: *(Study 4) Flowchart of Experimental Procedure*

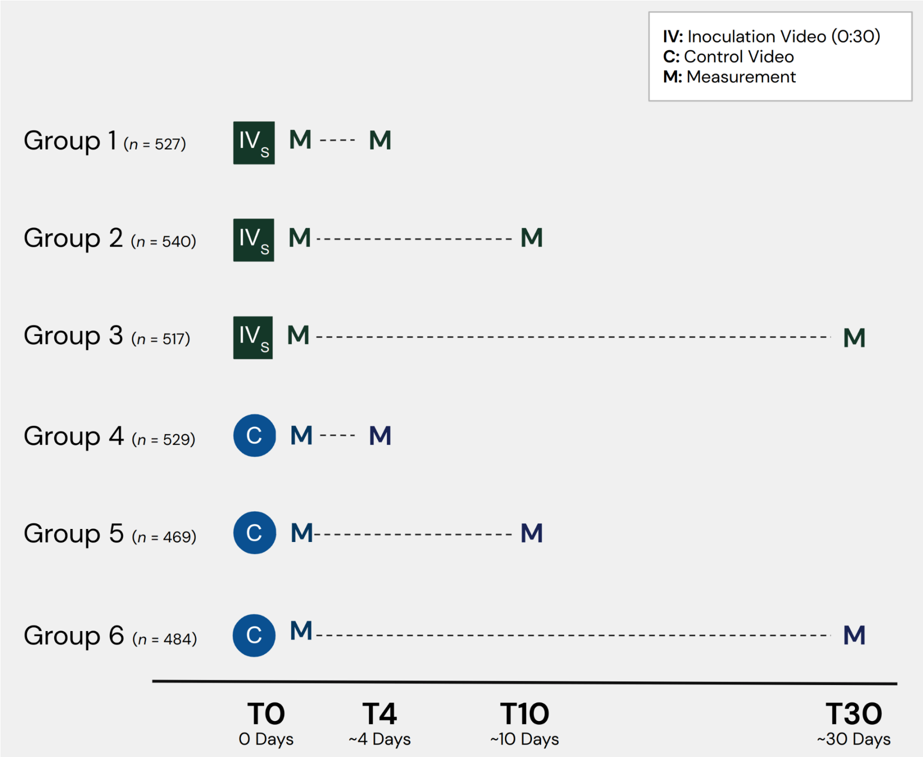

This figure depicts the flow of the experiment for each of the three experimental groups and for the three control groups in Study 4.

Supplementary Figure 24: *(Study 5) Flowchart of Experimental Procedure*

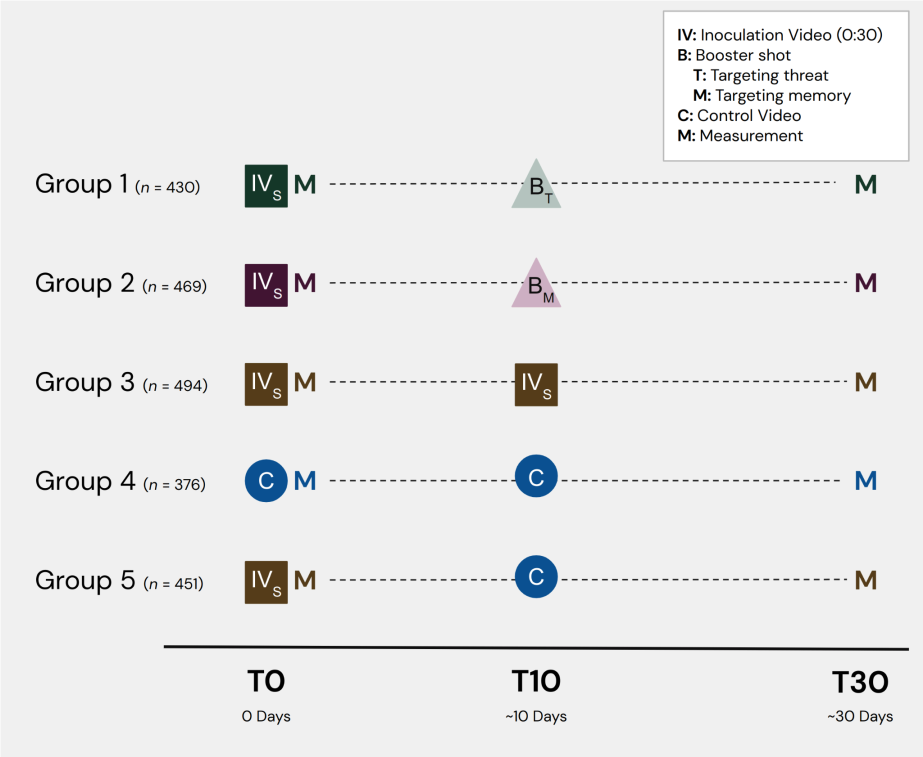

This figure depicts the flow of the experiment for each of the three experimental groups and for the two control groups in Study 5.

Supplementary Tables

Supplementary Table 1: *(Study 1) Overview of Preregistered Hypotheses*

#	Hypothesis	Evidence	Description
**H1**	Exposure to misinformation about climate change in the form of a false petition decreases the perceived scientific consensus (PSC) on global warming.	**YES**	**The misinformation effect was significant.**
**H2**	Inoculated individuals do not negatively change their perceived scientific consensus (PSC) on global warming after exposure to a misinformation message in the form of a false petition.	**YES**	**The inoculation effect was significant.**
**H3**	The inoculation effect described in H2 remains significant for at least 10 days (T10).	**YES**	**The inoculation effect was still significant after 10 days.**
**H4**	The inoculation effect described in H2 is no longer significant after 30 days (T30).	**INCONCLUSIVE**	**The inoculation effect was still significant after 30 days and therefore more robust than hypothesized, but the Bayesian analysis showed only anecdotal support (BF_{10} = 2.061).**
**H5**	The inoculation effect described in H2 is still significant after 30 days (T30), when individuals are exposed to a second inoculation message after 10 days (T10).	**YES**	**The boosted inoculation effect was significant after 30 days.**
**H6**	Groups exposed to a second inoculation message after 10 days (T10) show increased memory of the inoculation intervention after 30 days (T30) compared to those exposed to only one inoculation message.	**YES**	**The booster intervention improved memory.**
**H7**	Groups exposed to a second inoculation message after 10 days (T10) show increased motivational threat after 30 days (T30) compared to those exposed to only one inoculation message.	**NO**	**The booster intervention did not increase motivation.**
**H8**	The inoculation effect [H8a] immediately after intervention (T0), [H8b] after 10 days (T10), and [H8c] after 30 days (T30) is influenced directly by memory and motivation, and indirectly by the inoculation intervention (mediated by memory and motivation).	**YES**	**Across the time points, both the indirect inoculation effect and the direct effect of memory and motivation were significant.**

Note. See the main text for the full analyses, including the exact *p*-values and an alternative Bayesian analysis.

Supplementary Table 2: *(Study 1) Memory-Motivation Model Estimates*

Effect	*z*	*p*	β	95% CI		*SE*
				LL	*UL*	
Indirect						
**Inoc.T0 ⇒ Memory.T30 ⇒ PSC.T30**	**6.323**	**< .001**	**0.237**	**0.164**	**0.311**	**0.038**
Inoc.T0 ⇒ Motivation.T30 ⇒ PSC.T30	0.779	.436	0.019	-0.029	0.068	0.025
Inoc.T0 ⇒ Motivation.T30 ⇒ Memory.T30 ⇒ PSC.T30	0.744	.457	0.002	-0.003	0.006	0.002
Component						
**Inoc.T0 ⇒ Memory.T30**	**10.634**	**< .001**	**0.816**	**0.665**	**0.966**	**0.077**
**Memory.T30 ⇒ PSC.T30**	**7.864**	**< .001**	**0.291**	**0.218**	**0.363**	**0.037**
Inoc.T0 ⇒ Motivation.T30	0.782	.434	0.065	-0.098	0.228	0.083
**Motivation.T30 ⇒ PSC.T30**	**8.632**	**< .001**	**0.296**	**0.229**	**0.363**	**0.034**
**Motivation.T30 ⇒ Memory.T30**	**2.509**	**.012**	**0.088**	**0.019**	**0.157**	**0.035**
Direct						
Inoc.T0 ⇒ PSC.T30	0.945	.345	0.076	-0.082	0.233	0.080
Total						
**Inoc.T0 ⇒ PSC.T30**	**4.063**	**< .001**	**0.334**	**0.173**	**0.495**	**0.082**

Note. This table represents the results of the SEM analysis in Study 1 with two-sided *z*-tests, standardized β estimates, and 95% confidence intervals (CIs). Significant effects are presented in bold. $N = 689$.

Supplementary Table 3: *(Study 1) Dominance Analysis at T30*

Variable	Dominance
Memory	82%
Issue Involvement	4%
Issue Accessibility	4%
Apprehensive Threat	4%
Motivational Threat	2%
Self-Reported Remembrance	1%
Fear	1%
Issue Talk	1%

Note. This table shows the results of the dominance analysis at T30 in Study 1. $N = 689$.

Supplementary Table 4: *(Study 2) Overview of Preregistered Hypotheses*

#	Hypothesis	Evidence	Description
**H1**	People who complete a gamified inoculation intervention (Bad News) rate misleading social media posts as less reliable than people who complete a control task (Tetris).	**YES**	**The inoculation effect was significant.**
**H2**	The inoculation effect described in H1 remains significant for at least 10 days (T10).	**NO**	**The inoculation effect after 10 days was not significant, although it descriptively moved in the predicted direction ($p < .10$).**
**H3**	The inoculation effect described in H1 is no longer significant after 30 days (T30).	**INCONCLUSIVE**	**The inoculation effect was no longer significant 30 days after the intervention, but with only anecdotal support for the null hypothesis.**
**H4**	The inoculation effect described in H1 is still significant after 30 days (T30), when individuals participate in a booster intervention after 10 days (T10).	**INCONCLUSIVE**	**The boosted inoculation effect after 30 days was not significant, although descriptively moving in the predicted direction ($p < .10$), and with only anecdotal support for the null hypothesis (BF_{10} = 0.365).**
**H5**	People participating in a booster intervention after 10 days (T10) show increased memory of the inoculation intervention after 30 days (T30) compared to the control group.	**YES**	**The booster significantly improved memory.**
**H6**	People participating in a booster intervention after 10 days (T10) show increased motivational threat after 30 days (T30) compared to the control group.	**NO**	**The booster did not significantly improve motivation.**
**H7**	The inoculation effect [H7a] immediately after intervention (T0), [H7b] after 10 days (T10), and [H7c] after 30 days (T30) is influenced directly by memory and motivation, and indirectly by the inoculation intervention (mediated by memory and motivation).	**YES**	**Across the time points, both the indirect inoculation effect and the direct effect of memory and motivation were significant.**

Note. See the main text for the full analyses, including the exact *p*-values and an alternative Bayesian analysis.

Supplementary Table 5: *(Study 2) Memory-Motivation Model Estimates*

Effect	*z*	*p*	β	95% CI		*SE*
				LL	*UL*	
Indirect						
**Inoc.T0 ⇒ Memory.T0 ⇒ Fake.T0**	**-4.898**	**< .001**	**-0.548**	**-0.767**	**-0.329**	**0.112**
Inoc.T0 ⇒ Motivation.T0 ⇒ Fake.T0	-0.452	.651	-0.005	-0.028	0.018	0.012
Inoc.T0 ⇒ Motivation.T0 ⇒ Memory.T0 ⇒ Fake.T0	-0.454	.650	-0.002	-0.009	0.006	0.004
Component						
**Inoc.T0 ⇒ Memory.T0**	**17.564**	**< .001**	**1.472**	**1.308**	**1.636**	**0.084**
**Memory.T0 ⇒ Fake.T0**	**-5.100**	**< .001**	**-0.372**	**-0.515**	**-0.229**	**0.073**
Inoc.T0 ⇒ Motivation.T0	0.466	.641	0.055	-0.176	0.287	0.118
Motivation.T0 ⇒ Fake.T0	-1.853	.064	-0.097	-0.199	0.006	0.052
**Motivation.T0 ⇒ Memory.T0**	**2.133**	**.033**	**0.085**	**0.007**	**0.163**	**0.040**
Direct						
Inoc.T0 ⇒ Fake.T0	0.249	.803	0.038	-0.262	0.338	0.153
Total						
**Inoc.T0 ⇒ Fake.T0**	**-4.503**	**< .001**	**-0.517**	**-0.741**	**-0.292**	**0.115**

Note. This table represents the results of the SEM analysis in Study 2 with two-sided *z*-tests, standardized β estimates, and 95% confidence intervals (CIs). Significant effects are presented in bold. $N = 319$.

Supplementary Table 6: *(Study 2) Dominance Analysis at T30*

Variable	Dominance
Memory	60%
Motivational Threat	17%
Issue Talk	14%
Issue Accessibility	7%
Self-Reported Remembrance	1%
Issue Involvement	1%
Apprehensive Threat	1%
Fear	0%

Note. This table shows the results of the dominance analysis at T30 in Study 2. $N = 329$.

Supplementary Table 7: *(Studies 3–5) Overview of Preregistered Hypotheses*

#	Hypothesis	Evidence	Description
	**STUDY 3**		
**H1.1**	People who watched a short inoculation video (H1.1a) or a long inoculation video (H1.1b) are better at discerning manipulative social media posts from neutral social media posts than people who watched a control video.	**(Short Inoculation) YES**<br>**(Long Inoculation) YES**	**The inoculation effect was significant for both short and long inoculation videos.**
**H1.2**	The inoculation effect of a short inoculation video (0 min 30 sec) is smaller than the inoculation effect of a long inoculation video (1 min 48 sec).	**NO**	**The inoculation effects did not differ significantly between the short and the long videos, and the Bayesian analysis showed moderate evidence for the null hypothesis.**
**H1.3**	The inoculation effect of a long inoculation video (H1.3a) or a short inoculation video (H1.3b) decays partially but not completely over a period of two weeks.	**(Short Inoculation) INCONCLUSIVE**<br>**(Long Inoculation) INCONCLUSIVE**	**The inoculation effects were no longer significant after two weeks, but the Bayesian analyses showed only anecdotal evidence for the null hypothesis.**
	**STUDY 4**		
**H2.1**	People who watched an inoculation video are better at discerning manipulative social media posts from neutral social media posts than people who watched a control video.	**YES**	**The inoculation effect was significant.**
**H2.2**	There is no decay of the inoculation effect of inoculation videos after 4 days (T4).	**YES**	**The inoculation effect was still significant after 4 days.**
**H2.3**	There is partial decay of the inoculation effect of inoculation videos after 10 days (T10).	**NO**	**The inoculation effect did not show partial decay.**
**H2.4**	There is full decay of the inoculation effect of inoculation videos after 30 days (T30).	**INCONCLUSIVE**	**The inoculation effect was no longer significant after 30 days, but was descriptively moving in the direction of an effect and the Bayesian analysis showed anecdotal support for the alternative hypothesis (BF_{10} = 1.321).**
**H2.5**	Memory (forgetting) predicts inoculation decay.	**YES**	**Memory was a significant predictor of effect retention.**
**H2.6**	Threat (motivation) does not predict inoculation decay.	**NO**	**Motivation was a significant predictor of effect retention.**
	**STUDY 5**		
**H3.1**	People who watched an inoculation video are better at discerning manipulative social media posts from neutral social media posts than people who watched a control video.	**YES**	**The inoculation effect was significant.**

**H3.2**	The inoculation effect of inoculation videos is no longer significant after 30 days.	**NO**	**The inoculation effect was stronger than hypothesized.**
**H3.3**	An inoculation video (T0) that is followed by a threat-based booster video 10 days later (T10), is effective at keeping the inoculation effect significant up to 30 days after the T0 inoculation.	**YES**	**The threat-boosted inoculation effect was significant.**
**H3.4**	An inoculation video (T0) that is followed by a memory-based booster video 10 days later (T10), is effective at keeping the inoculation effect significant up to 30 days after the T0 inoculation.	**YES**	**The memory-boosted inoculation effect was significant.**
**H3.5**	An inoculation video (T0) that is followed by the same inoculation video 10 days later (T10), is effective at keeping the inoculation effect significant up to 30 days after the T0 inoculation.	**YES**	**The boosted inoculation effect was significant.**
**H3.6**	Groups exposed to a threat-based booster video at T10 show increased motivation (a), but not memory (b) of the intervention, at T30, compared to those inoculated who did not receive a booster video.	**(Memory) YES (null)**<br>**(Motivation) NO (null)**	**The threat-based booster did not significantly increase motivation nor memory.**
**H3.7**	Groups exposed to a memory-based booster video at T10 show increased memory (a) of the inoculation intervention, but not motivation (b), at T30, compared to those inoculated who did not receive a booster video.	**(Memory) YES**<br>**(Motivation) INCONCLUSIVE**	**The memory booster significantly improved memory but not motivation, although evidence for the null hypothesis for motivation was only anecdotal.**
**H3.8**	Groups exposed to a repeated-inoculation booster video at T10 show increased memory (a) of the inoculation intervention and motivation (b) at T30, compared to those inoculated who did not receive a booster video.	**(Memory) YES**<br>**(Motivation) INCONCLUSIVE**	**The re-inoculation booster significantly improved memory but not motivation.**
**H3.9**	The inoculation effect at T0 (a) and T30 (b) is influenced directly by memory and motivation, and indirectly by the inoculation intervention (mediated by memory and motivation).	**(T0) YES**<br>**(T30) YES**	**Across the time points, both the indirect inoculation effect and the direct effect of memory and motivation were significant.**

Note. See Supplementary Note 5 (Study 3), Supplementary Note 6 (Study 4), and the main text (Study 5) for the full analyses, including the exact *p*-values and an alternative Bayesian analysis.

Supplementary Table 8: *(Study 4) Memory-Motivation Model Estimates*

Effect	*z*	*p*	β	95% CI		*SE*
				LL	*UL*	
Indirect						
**Inoc.T0 ⇒ Memory.T4/T10/T30 ⇒ Discernment.T4/T10/T30**	**7.638**	**< .001**	**0.197**	**0.147**	**0.248**	**0.026**
**Inoc.T0 ⇒ Motivation.T4/T10/T30 ⇒ Discernment.T4/T10/T30**	**3.136**	**.002**	**0.017**	**0.006**	**0.028**	**0.005**
**Inoc.T0 ⇒ Motivation.T4/T10/T30 ⇒ Memory.T4/T10/T30 ⇒ Discernment.T4/T10/T30**	**2.576**	**.010**	**0.001**	**0.000**	**0.002**	**0.001**
Component						
**Inoc.T0 ⇒ Memory.T4/T10/T30**	**39.974**	**< .001**	**1.167**	**1.110**	**1.224**	**0.029**
**Memory.T4/T10/T30 ⇒ Discernment.T4/T10/T30**	**7.782**	**< .001**	**0.169**	**0.127**	**0.212**	**0.022**
**Inoc.T0 ⇒ Motivation.T4/T10/T30**	**3.420**	**< .001**	**0.123**	**0.053**	**0.194**	**0.036**
**Motivation.T4/T10/T30 ⇒ Discernment.T4/T10/T30**	**7.853**	**< .001**	**0.138**	**0.104**	**0.173**	**0.018**
**Motivation.T4/T10/T30 ⇒ Memory.T4/T10/T30**	**4.531**	**< .001**	**0.066**	**0.038**	**0.095**	**0.015**
Direct						
Inoc.T0 ⇒ Discernment.T4/T10/T30	1.073	.283	0.047	-0.038	0.131	0.043
Total						
**Inoc.T0 ⇒ Discernment.T4/T10/T30**	**7.322**	**< .001**	**0.262**	**0.192**	**0.333**	**0.036**

Note. This table represents the results of the SEM analysis in Study 4 with two-sided *z*-tests, standardized β estimates, and 95% confidence intervals (CIs). Significant effects are presented in bold. *N* = 3,066.

Supplementary Table 9: *(Study 5) Memory-Motivation Model Estimates*

Effect	*z*	*p*	β	95% CI *LL*	95% CI *UL*	*SE*
Indirect						
BoosterA.T10 ⇒ Motivation.T30 ⇒ Discernment.T30	1.149	.251	0.009	-0.006	0.024	0.008
Inoc2.T10 ⇒ Motivation.T30 ⇒ Discernment.T30	0.835	.403	0.006	-0.008	0.020	0.007
**Inoc2.T10 ⇒ Memory.T30 ⇒ Discernment.T30**	**5.567**	**< .001**	**0.074**	**0.048**	**0.099**	**0.013**
**BoosterB.T10 ⇒ Memory.T30 ⇒ Discernment.T30**	**4.883**	**< .001**	**0.064**	**0.038**	**0.089**	**0.013**
**Inoc1.T0 ⇒ Motivation.T0 ⇒ Motivation.T30 ⇒ Discernment.T30**	**2.345**	**.019**	**0.011**	**0.002**	**0.020**	**0.005**
**Inoc1.T0 ⇒ Memory.T0 ⇒ Memory.T30 ⇒ Discernment.T30**	**10.497**	**< .001**	**0.228**	**0.186**	**0.271**	**0.022**
**Inoc1.T0 ⇒ Motivation.T0 ⇒ Memory.T0 ⇒ Memory.T30 ⇒ Discernment.T30**	**2.129**	**.033**	**0.001**	**0.000**	**0.003**	**0.001**
Component						
BoosterA.T10 ⇒ Motivation.T30	1.162	.245	0.056	-0.039	0.152	0.049
**Motivation.T30 ⇒ Discernment.T30**	**7.764**	**< .001**	**0.156**	**0.117**	**0.196**	**0.020**
Inoc2.T10 ⇒ Motivation.T30	0.840	.401	0.039	-0.052	0.129	0.046
**Inoc2.T10 ⇒ Memory.T30**	**6.316**	**< .001**	**0.284**	**0.196**	**0.372**	**0.045**
**Memory.T30 ⇒ Discernment.T30**	**11.787**	**< .001**	**0.259**	**0.216**	**0.302**	**0.022**
**BoosterB.T10 ⇒ Memory.T30**	**5.365**	**< .001**	**0.245**	**0.156**	**0.335**	**0.046**
**Inoc1.T0 ⇒ Motivation.T0**	**2.471**	**.013**	**0.140**	**0.029**	**0.250**	**0.056**
**Motivation.T0 ⇒ Motivation.T30**	**26.356**	**< .001**	**0.488**	**0.452**	**0.525**	**0.019**
**Inoc1.T0 ⇒ Memory.T0**	**40.009**	**< .001**	**1.718**	**1.634**	**1.802**	**0.043**
**Memory.T0 ⇒ Memory.T30**	**28.238**	**< .001**	**0.513**	**0.477**	**0.549**	**0.018**
**Motivation.T0 ⇒ Memory.T0**	**4.546**	**< .001**	**0.073**	**0.042**	**0.105**	**0.016**
Direct						
BoosterA.T10 ⇒ Discernment.T30	0.017	.986	0.001	-0.124	0.126	0.064
Inoc2.T10 ⇒ Discernment.T30	-1.095	.274	-0.068	-0.189	0.054	0.062
**BoosterB.T10 ⇒ Discernment.T30**	**2.002**	**.045**	**0.126**	**0.003**	**0.248**	**0.063**
Inoc1.T0 ⇒ Discernment.T30	-0.652	.515	-0.045	-0.180	0.090	0.069
Total						
BoosterA.T10 ⇒ Discernment.T30	0.416	.677	0.028	-0.103	0.159	0.067
Inoc2.T10 ⇒ Discernment.T30	0.297	.766	0.019	-0.107	0.146	0.065
**BoosterB.T10 ⇒ Discernment.T30**	**3.179**	**.001**	**0.208**	**0.080**	**0.336**	**0.065**
**Inoc1.T0 ⇒ Discernment.T30**	**3.302**	**< .001**	**0.228**	**0.093**	**0.364**	**0.069**

Note. This table represents the results of the SEM analysis in Study 5 with two-sided *z*-tests, standardized β estimates, and 95% confidence intervals (CIs). Significant effects are presented in bold. $N = 2{,}220$.

Supplementary Table 10: *(Study 5) Dominance Analysis at T30*

Variable	Dominance
Memory	41%
Motivational Threat	27%
Fear	10%
Apprehensive Threat	9%
Issue Involvement	8%
Issue Talk	2%
Issue Accessibility	2%
Self-Reported Remembrance	0%

Note. This table shows the results of the dominance analysis at T30 in Study 5. $N = 2{,}220$.

Supplementary Methods

(Study 1) Information About the Climate Change Inoculation Paradigm

Although 97% of climate scientists agree that human-caused global warming is happening, misinformation sowing doubt about the consensus influences society [11,12]. Evidence suggests that debiasing public perception of the scientific consensus can lead to more support for collective action [13,14], but that this can be thwarted by misinformation [3,15]. In the seminal study by van der Linden et al. [3], participants were exposed to a consensus message in the form of a pie chart depicting the scientific consensus [6], an inoculation message warning people not to be convinced by false petitions and how this particular petition is flawed, and a misinformation message presenting the misleading Oregon Petition [7]. They found that communicating the actual scientific consensus helps, as it helped to debias the perceived scientific consensus (i.e., people correct their belief about the scientific consensus), but that misinformation can neutralize all benefits. They also found that an inoculation message was able to protect this benefit by significantly reducing the impact of the misinformation message. The outcome variable measured is the perceived scientific consensus on human-caused global warming, on a slider scale from 0%–100%. See Supplementary Fig. 15 for the consensus message (left) and the misinformation message (right). The following text represents the artificially created inoculation message that was shown to participants, adopted (entirely and unmodified) from van der Linden et al. [3] and Maertens et al. [4]:

Nearly all climate scientists—97%—have concluded that human-caused climate change is happening. Some politically-motivated groups use misleading tactics to try to convince the public that there is a lot of disagreement among scientists. However, scientific research has found that among climate scientists "there is virtually no disagreement that humans are causing climate change."

One such politically motivated group claims to have collected signatures from over 31,000 "scientists" (including over 9,000 who hold Ph.D.'s) on a petition urging the U.S. government to reject any limits on greenhouse gas emissions because; "there is no convincing scientific evidence that human release of carbon dioxide, methane or other greenhouse gases is causing or will, in the foreseeable future, cause catastrophic heating of the Earth's atmosphere and disruption of Earth's climate." They claim that these signatures prove that there is no scientific consensus on human-caused climate change.

This may sound convincing at first. However, several independent investigations have concluded that the "Oregon Petition" is extremely misleading. For instance, many of the signatures on the petition are fake (for example, past signatories have included the long-deceased Charles Darwin, members of the Spice Girls, and fictional characters from Star Wars). Also, although 31,000 may seem like a large number, it actually represents less than 0.3% of all US science graduates (a tiny fraction). Further, nearly all of the legitimate signers have no expertise in climate science at all. In fact, less than 1% of those who signed the petition claim to have any background in Climate or Atmospheric Science. Simply calling yourself a "scientist" does not make someone an expert in climate science. By contrast, 97% of actual climate scientists agree that human-caused climate change is happening.

In this paradigm, the inoculation message is passive, issue-specific, and therapeutic. Passive, as participants read the messages without interacting with them (i.e., the experimenter provides the counter-arguments for the participant to read and remember). Specific, as it targets only the perceived scientific consensus, and presents a tailored inoculation message that includes a weakened version of the particular misinformation message (i.e., the message is focused on countering a specific piece of climate misinformation). And therapeutic, as on average people have (inaccurate) pre-existing attitudes regarding the scientific consensus on human-caused global warming [3].

(Study 2) Information About the Bad News Inoculation Paradigm

In this inoculation intervention—which has already been played by over two million people and implemented in some school curricula in the United Kingdom and in Canada—the goal is to gain as many followers as possible by choosing and spreading misinformation messages while at the same time keeping your credibility sufficiently high. It includes the warning component of inoculation by showing the detrimental consequences misinformation can have (i.e., consequences of in-game actions) on topics that feel familiar (e.g., someone who gets fired from their job because of false accusations). This elicits a sense of threat and motivation to resist similar persuasion attempts (i.e., the game warns people, and this motivates them to protect themselves against misinformation).

Unique is that people are exposed to weakened doses of broader misinformation *techniques* rather than specific issues, making it broad-spectrum. In other words, if people are inoculated against an entire technique (e.g., conspiracy theorisation), they should gain resistance to different variants of that technique (e.g., different conspiracy theories). It uses a framework of six influential misinformation techniques known as DEPICT: Discrediting opponents (e.g., creating a cloud of doubt around your opponent), appealing to Emotion (e.g., the use of outrage or highly emotive language to manipulate people), Polarizing audiences (e.g., using hot-button issues to drive a wedge between two groups), Impersonation (e.g., misusing the identity of politicians, experts, or celebrities online), floating Conspiracy theories (e.g., casting doubt on mainstream narratives by providing an attractive story in which a small sinister group of people is responsible for doing harm to many), and Trolling (e.g., eliciting reactions from people by provoking them online). See Roozenbeek and van der Linden [8] and van der Linden and Roozenbeek [16] for a detailed background and overview of these techniques. The active thinking, content creation, and choices people make for each misinformation technique serves as the cognitive component of the inoculation (i.e., through

engaging with the weakened doses of misinformation, people generate preemptive refutations). This intervention serves as a broad-spectrum, active, and mainly prophylactic intervention. Broad, as it protects against a wide spectrum of different misinformation techniques (rather than specific misinformation messages). Active, as the intervention provides an experiential environment with interactive content. And mainly prophylactic, as the protection is aimed towards new information not seen before. See Supplementary Fig. 17 for an impression. Although we cannot know the players' prior level of exposure to the misinformation tactics when they enter the game, the content of the game is fictional and therefore it can be assumed that there was no prior exposure to the specific content presented. However, participants might have seen or believed some misinformation using these techniques before (e.g., conspiracy theories), and therefore it could be argued that it may also function in parallel as a therapeutic intervention.

It has been shown that Bad News is effective at making people detect misinformation [8,17]—replicated across cultures [18]—and at accurately increasing confidence in doing so [19]. Resistance is measured by letting participants judge the reliability of real news items (that are neutral, non-misleading, and non-manipulative) and fake news items (that use one of the DEPICT techniques) on a 7-point Likert scale (see Supplementary Fig. 18 for an example), before and after the Bad News intervention. Participants rate the fake news items as less reliable after the intervention, both compared to the pretest and compared to the control group [8]. However, as with the climate change paradigm, the long-term effectiveness of this paradigm has not been tested.

(Studies 3–5) Information About the Video-Based Inoculation Paradigm

In a research project between Google Jigsaw, the University of Cambridge, and the University of Bristol, inoculation researchers designed and tested five short videos (~90 seconds), each of which inoculates viewers against a manipulation technique commonly encountered in online environments: emotional language (fearmongering), incoherence, false dichotomies, scapegoating, and ad hominem attacks. See https://inoculation.science/inoculation-videos/ for an overview of the inoculation videos. In a first series of large randomized controlled trials ($N = 5,416$), the videos proved highly effective at 1) improving participants' ability to identify manipulation techniques in social media content; 2) increasing their confidence in their ability to spot such techniques; 3) strengthening their ability to discern trustworthy from untrustworthy content; and 4) improving the quality of their sharing decisions [10]. The videos are currently being rolled out as educational advertisements, and have been watched by over 5 million people. See Supplementary Fig. 20 for a screenshot of the emotional language video and Supplementary Fig. 21 for an example test item.

In this paradigm, participants watch a short inoculation video and subsequently rate 10 out of 20 possible social media posts (each participant receives the same 10 topics, but within each topic they have to rate either a manipulative or a non-manipulative variant of the item pair) on a 1–7 scale (1—strongly disagree, 7—strongly agree), for each of the following dimensions: Manipulativeness ("This post is manipulative"), Confidence ("I am confident in my assessment of this post's manipulativeness"), Trustworthiness ("This post is trustworthy"), Sharing Intention ("I would share this post with people in my network"). In addition to the 50% chance per topic of seeing the manipulative or non-manipulative variant, 5/10 topics contained only content based on actual social media sources (for both the manipulative and non-manipulative variants), and the other five topics used only fictive items

specifically created for this experiment (for both the manipulative and non-manipulative variants). It is therefore possible that there is an imbalance in manipulative compared to non-manipulative items presented, but the ratio of real compared to created items is always balanced. Subsequently, a discriminative ability index for manipulativeness, trustworthiness, and sharing is calculated by subtracting the average scores of neutral posts from the average scores for the manipulative posts. For the confidence measure, results for manipulative and neutral posts are reported separately.

The intervention can be seen as a broad-spectrum, passive, and mainly prophylactic intervention. Broad, as it focuses on a general technique (e.g., emotional language) and thus protects against a wider range of potential misinformation messages. Passive, as people watch a video without any possibility to interact. And mainly prophylactic, as it aims to protect people against messages and attacks they are not familiar with. Similar to the Bad News game intervention the content in the video is mostly fictional and therefore prior exposure should be limited, but some participants may be familiar with misinformation featuring the manipulative tactic (e.g., appeal to emotion). It therefore may function as both prophylactic and therapeutic inoculation.

Supplementary Discussion

Mapping and Boosting the Longevity of Counter-Misinformation Interventions

Maertens et al. [4] found that text-based, passive, therapeutic, issue-specific inoculation interventions are effective, replicating the results of previous research [3,15], and can remain fully effective for at least one week, while the effect of a consensus-message-based treatment without inoculation does not. This indicates that even when inoculation is passive, a strong initial effect can be established. In Study 1, we expanded on this study to test the memory-motivation model using objective and subjective measures of the model's components, and included longer timeframes (T30 *Mdn* = 29 days). The effect after week was in line with the meta-analytic [20] effect size of inoculation ($d_{\text{Study2}} = 0.46$ $d_{\text{MetaAnalysis}} = 0.43$), and reduced by 39% after one month ($d_{\text{0days}} = 0.46$, $d_{\text{8days}} = 0.38$, $d_{\text{29days}} = 0.28$). These studies indicate that the inoculation effect remains intact for at least 1 month without any booster intervention with text-based inoculation messages on climate change, but that light decay takes place. This is in line with findings by a recent study by Ivanov et al. [21], who reported significant decay after 6 weeks but not after 4 weeks, as well as the meta-analysis by Banas and Rains [20], that reported decay to typically start to take place after 2 weeks. Meanwhile, we found the first evidence that a booster intervention—in this case a repetition of the original inoculation message—can boost the effect to prevent any decay from happening, in particular by boosting memory of the intervention ($d_{\text{0days}} = 0.46$, $d_{\text{29days,NoBoost}} = 0.28$, $d_{\text{29days,Boost}} = 0.48$). This finding is in line with Ivanov et al. [21] who suggest that repeated inoculation messages could be effective at lengthening the inoculation effect. Investigating the underlying mechanisms showed that motivational threat and issue involvement are predictors of the outcome variable, but that objective memory of the inoculation intervention was the strongest predictor of the inoculation effect over time. We also found that motivation had a positive influence on inoculation memory, in line with the predictions of the memory-motivation

model. However, there was no evidence for an effect of the intervention on motivation. As we also found that motivation directly influenced the outcome variable, an important question for future research is whether we can improve our inoculation interventions in order to elicit more motivation [22,23].

In Maertens et al. [9], we investigated the same questions in a gamified, active, broad, prophylactic, inoculation paradigm. We found that repeated testing can serve as an inoculation booster, allowing for the inoculation to remain fully intact for up to 3 months, potentially due to memory strengthening that comes with testing. However, when not testing repeatedly, the effect was no longer significant after 2 months. The study also showed that these findings were not due to item or item ratio effects. In Study 2, we expanded on this study by removing the repeated testing confound and splitting the sample in groups per posttest time point, and adding the same set of questions about memory and motivation. we replicated the main effect of the Bad News game, with a larger effect (d_{Study4} = 0.78, $d_{\text{MetaAnalysis}}$ = 0.43) than the typical effect size found in inoculation interventions [20], but also found that when an immediate posttest is not included, the inoculation effect is no longer significant after 9 days, a faster effect decay than anticipated. When looking into the mechanisms, memory arises as the most dominant predictor, followed by motivational threat. Meanwhile, those high in memory did show an inoculation effect at 9 days and at 29 days, and a new and short version of the Bad News presented after 9 days worked well as a memory booster—although not enough to show a general inoculation effect after 29 days. A test of the memory-motivation model revealed new insights that are different from the climate change paradigm. In this paradigm, only memory was a significant predictor of the outcome measure. Motivation did not have an influence on the outcome measure, nor did the inoculation intervention have an effect on motivation. However, motivation on its own did have a significant effect on memory formation at T0. In line with the findings from the climate

change paradigm, we found evidence for a full mediation of the inoculation effect through memory. These findings provide a second evidence base for the memory-motivation model, and evokes similar questions about the role of motivation in inoculation paradigms [22,23]: is it less important than previously thought, or did we fail to capture or move it appropriately?

In Study 3, we explored the same questions in a scalable, video-based, passive, broad, prophylactic form of inoculation. The study shows that both a short and long inoculation video can serve as an effective inoculation intervention, and that they are similarly effective, with an effect size ($d_{LongVideo} = 0.53$, $d_{ShortVideo} = 0.44$, $d_{MetaAnalysis} = 0.43$) similar to the meta-analytic effect size of inoculation interventions [20]. When no immediate posttest is included, we can see a quick inoculation effect decay after the intervention in the course of the first two weeks, paralleled by a similar decay curve for memory. However, when an immediate posttest is implemented, the video inoculation effect can remain effective for up to at least 29 days, with limited decay ($d_{Exp3,0days} = 0.30$, $d_{Exp3,29days} = 0.23$). When investigating the mechanisms, memory was again the most dominant predictor of the inoculation outcome. In addition, to disentangle the mechanisms further and explore the potential of booster interventions, we tested three booster videos, a repetition of the original video, a threat-focused booster, and a technique memory booster. Both the memory booster video and the repeated original inoculation video served successfully to boost inoculation memory, while the threat booster video did not impact memory performance and failed to increase motivation. Similar to Study 1 and Study 2, both memory and motivation were significant predictors of the inoculation effect. However, this time the original intervention did have a direct positive impact on motivation as well, which could mean that different types of inoculation interventions work through different mechanisms. Different from the previous two tests of the memory-motivation model, we now were able to disentangle T0 and T30 effects, and found that the intervention successfully improved memory and motivation at T0,

and that motivation improved memory at T0, which in turn increased the inoculation effect at T30 through memory at T30, in line with the memory-motivation model predictions.

The studies in this paper represent a first look at developing a memory paradigm for inoculation. Many of the choices made in this work, including the choice of measures for memory and motivation, the causal ordering of the SEM models, and the used inoculation interventions, mean that the validity of the model needs to be thoroughly and independently tested before it can become the new standard. Nevertheless, taken together, these first direct measures of the mechanisms behind the longevity of the inoculation effect, explored across 5 studies (9 experiments) and 3 paradigms, suggest that a memory-motivation theory is a new feasible paradigm to consider and explore further. It also helps to formulate a data-driven answer to the main research question presented at the beginning of the paper: can we explain the resistance to persuasion decay process using a memory-motivation theory of inoculation decay? Not only did we find evidence for a role of memory in the explanation of the inoculation effects, the data suggests that it presents a better explanation of the longevity of the effects than the traditional account based on threat and motivation [24–31]. We do not find evidence for the need for an endured sense of threat for the inoculated persons to defend themselves against misinformation attacks at later time points, although we do find some evidence that motivation helps and that it could improve how much people learned from the inoculation intervention. In other words, these findings provide crucial new insights into resistance to persuasion. While threat and motivation have often been mentioned as a crucial aspect of inoculation, and proposed to be elicited as part of the affective forewarning in inoculation messages, literature studies show that the original authors and the first generation of inoculation scholars often did not manipulate nor measure it [22–24], although recently more research has been done that establishes its role [32–34]. In the text-based and gamified interventions we did not manage to manipulate motivational threat through our inoculation

intervention, and therefore might have missed a crucial aspect of what constitutes an inoculation intervention, but we did manage to do so in the video-based intervention. All studies in this paper are congruent however in their finding that motivation can have a role, but that the role of memory is typically larger. Therefore, the data in this work provides a strong basis for an alternative theory positing that inoculation can be modeled as memory networks and trained accordingly [35]. Although Pfau et al. [35] were not arguing against threat as much as arguing that memory and mental association were additional elements of the inoculation process (i.e., it was not considered by them to be competing processes or explanations), the evidence in this paper would suggest that although threat has an important role in the inoculation process, the memory processes are more essential (for longevity) than the threat processes. This opens up new possibilities to integrate insights from cognitive psychology related to learning, memory strengthening, and forgetting into inoculation theory [36–42]. The model proposed in the Introduction of this paper provides an example of such a model. Although promising, it has to be taken into account that this is a primitive first version of the model and needs to be replicated and tested in different forms in future research. It is for example possible that there are important aspects of inoculation interventions that moderate the relationships between the variables in the model. Some interventions may for example work by manipulating motivational threat in a different way (e.g., some inoculation interventions may work via memory, others via motivation, and others by manipulating both). There are also many alternative SEM models that could theoretically be viable instead of the currently used one, for example with a different causal ordering (e.g., it is possible that memory at T0 influences motivation at T0 instead of the other way around). Future research will need to disentangle these mechanisms and effects further.

Integrating Memory Research and Inoculation Theory

Not only does the new memory-based approach present theoretical relevance, it also presents a practical benefit for intervention developers and policy makers, as we can now start to form an answer to the empirical question of this hypothesis: what is the shape of the inoculation effect decay curve and can booster interventions remediate the decay? Plotting the data from all three paradigms together—which can be seen in Supplementary Fig. 14—we do indeed find a decay curve that resembles an exponential function, what one would expect when looking at an Ebbinghaus forgetting curve [37,40]. The below figure depicts the inoculation effect curve over time for each of the interventions in the first column, taken from Study 1, Study 2, and Study 3, and their respective memory forgetting curve in the second column. For the plot of Study 3 we combined the datasets of the three experiments, taking the control group and single inoculation group from Experiment 1, and grouping all second posttests from the inoculation groups from Experiments 2–3 as booster groups due to repeated testing and the various booster interventions. To simplify the plot the time points were grouped based on the median days after T0 (i.e., T10 = 9 days, T30 = 29 days). As can be seen, the decay curves of the inoculation effects are remarkably similar to the forgetting curves of the inoculation memory. When taking into account that the memory measures were newly created for each study and had not been validated before, and that each of the inoculation interventions had both very different modes of presentation (text-based, gamified, video-based) as well as very different outcome measures (perceived scientific consensus, reliability rating of fake news, and discernment of manipulativeness), this congruence is a promising first step towards unveiling the true decay curve of inoculation effects. In panel A and panel B, we can see that there is a strong inoculation effect for text-based inoculation as well as a strong memory, but that they, in a similar fashion, decay over time in what closely resembles an exponential curve, with some memory and some inoculation effect remaining

after 29 days. When boosted almost the full inoculation effect together with the full inoculation memory remains. A very similar pattern can be found for the gamified intervention in panel C and panel D. Despite the effect no longer being significant after 9 days, we do see the congruence between the inoculation effect and the memory forgetting curve. Finally, in the video-based intervention, as seen in panel E and F, we again find that the memory forgetting curve, as well as the booster curve, is closely mirroring the inoculation effect pattern. In addition, we find that after 1–2 weeks, the effect is no longer significant, but when boosted, the effect decays much slower than it decayed initially, and the same can be found for the inoculation memory. This shows what we would expect from a forgetting curve, namely, that once the memory is strengthened, the decay afterwards is slower [37,40]. The contrasts between the effectiveness of the interventions, and whether they are still significant after 1–2 weeks or not, could be explained by differing decay curves as well as by differing initial effectiveness. The results depicted here indicate that there is some uniformity in the decay curves across interventions, but also some variety in the rate of the decay. Whether this is a side effect of the different outcome measures or the small variations in the memory questions, or due to actual differences in effects or memory strength, will need to be explored in future research.

Conceptual and Methodological Issues in Longitudinal Inoculation

The study of the long-term effectiveness of inoculation effects should not be limited to the decay of resistance to persuasion, as Hill et al. [43, p. 532] stressed:

> *"Decay of the effects of political persuasion is too important to be ignored, as it routinely has been. It is a basic feature of mass persuasion in most if not all political contexts. Scholars should therefore try harder to build measurement of decay into their research designs."*

We would go further than this statement and stress that during the literature review for this paper, it became clear that important theories are often accepted with limited or no longitudinal research. This is not surprising: longitudinal research, especially with multiple timepoints and long-interval follow-ups, is both costly and difficult to run. A recent review of framing research highlights that long-term effects are often not measured, and that most longitudinal studies do not look beyond two weeks [44]. Similarly, in a meta-analysis of behavioral intervention studies regarding action on climate change, Nisa et al. [45, p. 9] stressed that they *"could not provide a definitive answer on persistent effects per specific type of intervention due to the small number of papers that reported follow-up effects"*. Within this work we developed and tested various formats of different longitudinal designs for each inoculation paradigm, with and without booster treatments. We looked beyond the standard single-treatment study and mapped long-term cognitive changes, thereby providing valuable insights relevant for the wider field of psychology, and inviting other researchers to consider a longitudinal design in their future studies as well.

During this journey, three important methodological questions—that were previously unanswered for inoculation research—were explored: item effects, testing effects, and psychometric validity. In Roozenbeek et al. [46], we looked at the potential confound of the

inoculation effect interpretations caused by the use of a pretest and the researcher's choice of items as the dependent variable for misinformation reliability ratings. The research showed that there is no evidence for an effect caused by the implementation of a pretest, but that the specific choice of items has an influence on the effect size found. In this case the effect remained significant despite the change in items, but recent research by Roozenbeek, Traberg, et al. [17] replicated the item effect and found that the effect can even change in direction when items are different for the pre and post tests. In other words, researchers have to be careful when choosing their items, and it stresses the need to work towards standardized item sets.

Despite there being no evidence for pretesting effects, the other studies in this work made clear that there are two other testing effects that we do need to take into account: the immediate posttest and repeated posttests. The small difference in design between Maertens et al. [9] (with immediate posttest) and Study 2 (without immediate posttest), and between Study 3 (without immediate posttest) and Studies 4–5 (with immediate posttest), demonstrated that the use of an immediate posttest potentially serves as an immediate memory booster. This is an important finding both for intervention implementation guidelines and for intervention evaluation science. It shows that an immediate posttest may not be advisable for evaluations of the long-term effectiveness evaluation of an intervention. While it could be argued that participants learn how to respond to particular items, we did not find evidence for this in Studies 3–5, where participants had to discern the manipulativeness of a random set of headlines from a larger pool of social media posts at each time point, with the possibility that items of the same topic switch from manipulative to neutral between time points. Similar to the immediate posttest effect, the difference in design between Maertens et al. [9], Experiment 1 (with immediate posttest and repeated posttests at multiple time points) and Maertens et al. [9], Experiment 2 (with immediate posttest but no additional repeated

posttests until the final time point), shows that repeating a posttest at multiple time points may serve as an additional booster on top of the immediate posttest. Also this finding fits into findings from the literature outside of the inoculation scholarship, in particular from cognitive psychology, with previous research finding similar learning effects by repeated testing [47–50]. Combined, the immediate posttest and the repeated posttest effects indicate that one should ideally use a design that exposes each participant to a maximum of one posttest (e.g., with each participant or group of participants receiving the posttest at a different point in time after the intervention, similar to Study 1, Study 2, and Study 3). This finding also has a positive side—it indicates that if practitioners are implementing an intervention in the field, it may be useful to consider including a quiz or a feedback mechanism at the end of the intervention to consolidate participants' knowledge, and repeatedly follow-up with the participants of the intervention over time, to further strengthen and increase the longevity of the effects.

Supplementary Notes

Supplementary Note 1: *(Study 1) SEM Model Estimates Analysis*

We found that, in line with the hypothesis, that there was a direct effect of inoculation memory on the PSC at T0, $z = 5.51$, $p < .001$, $\beta = 0.230$, 95% CI [0.148, 0.311], at T10 (8 days), $z = 7.93$, $p < .001$, $\beta = 0.316$, 95% CI [0.227, 0.406], and at T30 (29 days), $z = 7.86$, $p < .001$, $\beta = 0.291$, 95% CI [0.218, 0.363]. Similarly, a direct effect was found of motivation on the PSC at T0, $z = 4.77$, $p < .001$, $\beta = 0.177$, 95% CI [0.104, 0.250], T10, $z = 4.96$, $p < .001$, $\beta = 0.200$, 95% CI [0.121, 0.280], and at T30, $z = 2.51$, $p = .012$, $\beta = 0.088$, 95% CI [0.019, 0.157]. Meanwhile, the inoculation intervention had a direct influence on memory at T0, $z = 12.93$, $p < .001$, $\beta = 0.907$, 95% CI [0.769, 1.044], at T10, $z = 11.97$, $p < .001$, $\beta = 0.926$, 95% CI [0.774, 1.077], and at T30, $z = 10.63$, $p < .001$, $\beta = 0.816$, 95% CI [0.665, 0.966]. Also motivation had an impact on memory at T0, $z = 4.94$, $p < .001$, $\beta = 0.173$, 95% CI [0.105, 0.242], at T10, $z = 2.45$, $p = .014$, $\beta = 0.095$, 95% CI [0.019, 0.170], and at T30, $z = 2.51$, $p = .012$, $\beta = 0.088$, 95% CI [0.019, 0.157]. The intervention did not have a direct influence on motivation at T0, $z = 0.51$, $p = .608$, $\beta = 0.041$, 95% CI [-0.116, 0.199], at T10, $z = 1.26$, $p = .207$, $\beta = 0.111$, 95% CI [-0.061, 0.283], or at T30, $z = 0.78$, $p = 0.434$, $\beta = 0.065$, 95% CI [-0.098, 0.228]. Finally, the inoculation intervention had an indirect influence on the PSC mediated by memory at T0, [H8a] $z = 5.07$, $p < .001$, $\beta = 0.208$, 95% CI [0.128, 0.289], T10, [H8b] $z = 6.00$, $p < .001$, $\beta = 0.293$, 95% CI [0.197, 0.388], and at T30, [H8c] $z = 6.32$, $p < .001$, $\beta = 0.237$, 95% CI [0.164, 0.311], providing evidence in line with the memory-motivation model. While not preregistered, to investigate the nature of the mediation model further, we also looked at the direct effect of inoculation on the PSC at T30, and found that it was not significant $z = 0.95$, $p = .341$, $\beta = 0.076$, 95% CI [-0.082, 0.233], while the indirect effect was significant $z = 4.06$, $p < .001$, $\beta = 0.334$, 95% CI [0.173, 0.495]. This provides evidence for full mediation. See Supplementary Table 2 for a complete overview.

Supplementary Note 2: *(Study 1) Underlying Mechanisms Dominance Analysis*

Looking further into the mechanisms of the inoculation effect, setting out to find out what the strongest predictor is of the inoculation effect, we implement a dominance analysis with the T30 data of a wide range of predictors of the inoculation outcome mentioned in the literature. Dominance analysis is a method to investigate the relative importance of each predictor variable in a regression model by calculating the additional variance explained (R^2) of each variable in all possible model combinations with these variables and then performing pairwise comparisons for each of these subsets to establish which variable was more important (i.e., more dominant), leading to a percentage of the cases where one variable was dominant above the other variables [51]. This allowed us to identify which predictors were the most essential predictors. We use the T30 data as this time point is most relevant in terms of uncovering the mechanisms behind the long-term effectiveness. The analysis demonstrated that memory was by far the most dominant predictor of the inoculation effect (82%). See Supplementary Table 3 for a complete overview.

Supplementary Note 3: *(Study 2) SEM Model Estimates Analysis*

We found, in line with [H7], that memory had a direct influence on fake news reliability ratings at T0 [H7a], $z = -5.10$, $p < .001$, β = -0.372, 95% CI [-0.515, -0.229], at T10 [H7b], $z = -3.14$, $p = .002$, β = -0.225, 95% CI [-0.365, -0.084], and at T30 [H7c], $z = -4.16$, $p < .001$, β = -0.242, 95% CI [-0.355, -0.128]. However, motivational threat was not a significant predictor of fake news reliability ratings at T0 [H7a], $z = -1.85$, $p = .064$, β = -0.097, 95% CI [-0.199, 0.006], at T10 [H7b], $z = -0.15$, $p = .883$, β = -0.009, 95% CI [-0.133, 0.114], or at T30 [H7c], $z = -1.18$, $p = .238$, β = -0.064, 95% CI [-0.169, 0.042]. Motivation did significantly influence memory formation at T0, $z = 2.13$, $p = .033$, β = 0.085, 95% CI [0.007, 0.163], in line with the memory-motivation model.

Further in line with the memory hypothesis of H7, inoculation had an indirect effect on fake news detection outcome mediated through memory at T0 [H7a], $z = -4.90$, $p < .001$, β = -0.548, 95% CI [-0.767, -0.329], at T10 [H7b], $z = -2.94$, $p = .003$, β = -0.215, 95% CI [-0.358, -0.072], and at T30 [H7c], $z = -3.62$, $p < .001$, β = -0.192, 95% CI [-0.296, -0.088]. Although not preregistered, we also looked at whether the direct effect of the inoculation intervention was still significant at T0 when accounting for memory, and we found that the direct effect was no longer significant, $z = 0.25$, $p = .803$, β = 0.038, 95% CI [-0.262, 0.338].

See Supplementary Table 5 for a complete overview.

Supplementary Note 4: *(Study 2) Underlying Mechanisms Dominance Analysis*

We performed a dominance analysis on the possible predictors of the fake news reliability rating at T30 (see the methods section of Study 1 for an explanation of dominance analysis). We found that memory was the dominant predictor, followed by motivational threat. See Supplementary Table 6 for a complete overview.

In addition, although not preregistered, a Pearson correlation test reveals a significant negative correlation between memory and fake news reliability ratings in the inoculated groups, $t(564) = -8.69$, $p < .001$, $r = -.344$, 95% CI [-.414, -.269], as well as a significant negative correlation between memory and time, $t(564) = -5.77$, $p < .001$, $r = -.236$, 95% CI [-.312, -.157], similar to the positive correlation between fake news reliability ratings in the inoculation group and time, $t(564) = 3.94$, $p < .001$, $r = .164$, 95% CI [.082, .243].

Supplementary Note 5: *(Study 3) Evidence for Hypotheses Analysis*

To test our main hypotheses for the manipulativeness measure (i.e., manipulative language discernment), we preregistered a two-way (3x2) ANOVA analysis. We found that the omnibus test for the intervention is significant, $F(2, 2213) = 51.11, p < .001$, BF_{10} = 3.301e+9 (error = 0.027%), indicating that we can continue to test our contrasts as planned. As preregistered, we then conducted a series of Tukey-corrected ANOVA contrast tests to test hypotheses H1.1–H1.3. We found that the inoculation effect for the long inoculation video as compared to the control video is significant, $M_{\text{diff}} = 0.75$, $t(2213) = 7.43$, $p_{\text{tukey}} < .001$, d = 0.525, 95% CI [0.385, 0.664], BF_{10} = 1.002e+10 (error < 0.001%), providing evidence in line with H1.1a. Also the short inoculation video compared to the control video leads to a significant effect $M_{\text{diff}} = 0.63$, $t(2213) = 6.36$, $p_{\text{tukey}} < .001$, $d = 0.439$, 95% CI [0.303, 0.575], BF_{10} = 2.430e+7 (error < 0.001%), in line with H1.1b. The above analyses indicate significant medium effect sizes both for the long inoculation video and for the short inoculation video, replicating the original study [10], in favor of H1.1: both videos significantly improve participants' ability to discern manipulative from non-manipulative content. Now that the baseline effect is established, we can compare the short and the long inoculation videos and explore the decay over time.

We now test the contrast of the manipulative discernment scores after the short and long video. The videos did not show a significantly different effect from one another in terms of T0 effect sizes $M_{\text{diff}} = 0.12$, $t(2213) = 1.22$, $p_{\text{tukey}} = .826$, $d = 0.085$, 95% CI [-0.051, 0.222], BF_{10} = 0.158 (error = 0.126%), advising rejection of H1.2, indicating that the long and short videos are equally effective in the immediate post-test. Comparing the T12 (*Mdn* = 12 days after T0) and T0 decay in the long inoculation condition, we found that a significant decay takes place, $M_{\text{diff}} = -0.36$, $t(2213) = -3.43$, $p_{\text{tukey}} = .008$, $d = -0.255$, 95% CI [-0.400, -0.109], BF_{10} = 16.717 (error = 0.001%). Moreover, after this decay, the inoculation effect was no

longer significantly different from the control condition $M_{\text{diff}} = 0.24$, $t(2213) = 2.23$, $p_{\text{tukey}} = .227$, $d = 0.171$, 95% CI [0.020, 0.322], $BF_{10} = 0.998$ (error = 0.021%), although the evidence for the null hypothesis was only anecdotal. A similar result can be found when comparing T12 to T0 of the short inoculation videos $M_{\text{diff}} = -0.31$, $t(2213) = -2.90$, $p_{\text{tukey}} = .044$, $d = -0.216$, 95% CI [-0.362, -0.070], $BF_{10} = 5.391$ (error = 0.004%), and when comparing T12 short inoculation to T12 control $M_{\text{diff}} = 0.18$, $t(2213) = 1.58$, $p_{\text{tukey}} = .611$, $d = 0.124$, 95% CI [-0.030, 0.278], $BF_{10} = 0.332$ (error = 0.059%). These decay analyses indicate that there is full decay of the inoculation effect when measuring 12 days after T0, against H1.3 expectations, but also here the evidence is only anecdotal and thus inconclusive. See Supplementary Fig. 5 for a visual plot of manipulativeness discernment (Panel A) and memory (Panel B) over time. Although not preregistered, we also ran the above analyses with the confidence, trustworthiness, and sharing intent measures. Here, similar to the analyses for manipulativeness, we found significant effects for T0 (each in the expected direction), and significant decay to the extent that the effect is no longer significant when the Tukey *p*-value correction is administered, except for trustworthiness discernment in the long video. A larger sample would be needed to determine the presence of a reduced effect. All effects were driven by the scores for the manipulative items, with minimal change for non-manipulative items.

Supplementary Note 6: *(Study 4) Evidence for Hypotheses Analysis*

As preregistered, we tested hypothesis H2.1 by running an ANOVA with manipulativeness discernment as the dependent variable and group (inoculated or not) as the independent variable, with the full T0 dataset (N = 4,821). To test H2.1, we looked at the main effect of the intervention at T0 and found that the inoculation effect is significant, M_{diff} = 0.47, $t(4819) = 11.61$, $p_{tukey} < .001$, $d = 0.334$, 95% CI [0.278, 0.391], BF_{10} = 1.927e+27 (error < 0.001%).

To test the decay hypotheses H2.2, H2.3, and H2.4, we made use of an ANOVA with posttest discernment as a dependent variable and group and evaluation date as independent variables. In addition, we now used all time points, and only include data from participants who completed the follow-up within 3 days from the intended follow-up date (N = 3,066, $Mdn_{BetweenDays,T4} = 4$, $Mdn_{BetweenDays,T10} = 8$, $Mdn_{BetweenDays,T30} = 29$). The omnibus test for group was significant, $F(1, 3060) = 53.33$, $p < .001$, BF_{10} = 1.244e+10 (error < 0.001%). In line with our expectations, we found evidence for the stability of the effect over 4 days, with a significant effect compared to the control group, $M_{diff} = 0.53$, $t(3060) = 6.10$, $p_{tukey} < .001$, d = 0.375, 95% CI [0.254, 0.496], BF_{10} = 3.121e+6 (error < 0.001%), and no significant change in the inoculation groups between the two time points, $M_{diff} = 0.18$, $t(6124) = 2.54$, p_{tukey} = .178, $d = 0.128$, 95% CI [0.029, 0.226], $BF_{10} = 0.102$ (error = 0.029%). After 8 days we found that the effect was still significant compared to the control group, $M_{diff} = 0.41$, $t(3060)$ = 4.56, $p_{tukey} < .001$, $d = 0.288$, 95% CI [0.164, 0.412], $BF_{10} = 1645.807$ (error < 0.001%), and—contrary to our expectations—that there was no significant change between T0 and T10 in the inoculation groups, $M_{diff} = 0.04$, $t(6124) = 0.54$, $p_{tukey} > .999$, $d = 0.027$, 95% CI [-0.070, 0.125], $BF_{10} = 0.015$ (error = 0.032%). After 29 days we found that, in line with our preregistered hypothesis, that the inoculation effect is no longer significant compared to the control group, $M_{diff} = 0.18$, $t(3060) = 2.05$, $p_{tukey} = .315$, $d = 0.130$, 95% CI [0.006, 0.254],

BF_{10} = 1.321 (error = 0.016%), although the Bayesian analysis showed anecdotal evidence towards an effect, and there was no significant decay in the inoculation group when comparing T30 to T0, $M_{diff} = -0.01$, $t(6124) = 2.05$, $p_{tukey} > .999$, $d = -0.010$, 95% CI [-0.109, 0.089], BF_{10} = 0.005 (error = 0.033%). See Supplementary Fig. 6 for a plot of manipulativeness discernment (Panel A) and memory (Panel B) over time.

To test H2.5 and H2.6 and compare the mechanisms with the results from Study 1 and Study 2, we modeled an SEM model using the lavaan package in R [52] with second posttest memory and motivational threat as mediators for the manipulativeness discernment at second posttest, and T0 inoculation as the predictor variable, allowing direct effects from inoculation to memory, motivational threat, and discernment, and direct effects from memory and motivational threat to discernment. See Supplementary Fig. 7 for a schematic visualization of the model and its direct and indirect relationships, and Supplementary Table 8 for its model estimates. As predicted, we found that memory directly predicts the inoculation effect at a later time point, $t(3062) = 7.78$, $p < .001$, $\beta = 0.169$, 95% CI [0.126, 0.212], as did motivation, $t(3062) = 7.85$, $p < .001$, $\beta = 0.138$, 95% CI [0.104, 0.173]. All indirect and all component effects were significant with a significant total effect of the inoculation intervention, $t(3062) = 7.32$, $p < .001$, $\beta = 0.262$, 95% CI [0.192, 0.333], and no significant direct effect of the intervention, $t(3062) = 1.07$, $p = .238$, $\beta = 0.047$, 95% CI [-0.038, 0.131], providing evidence for full mediation.

Supplementary Note 7: *(Study 5) SEM Model Estimates Analysis*

We found evidence for partial mediation at T0, with memory, $b = 0.22$, $t(2216) = 13.24$, $p < .001$, $\beta = 0.351$, 95% CI [0.299, 0.403], and motivation, $b = 0.08$, $t(2216) = 3.89$, $p < .001$, $\beta = 0.079$, 95% CI [0.039, 0.118], having an effect on manipulativeness discernment, but meanwhile keeping intact a direct effect of inoculation, $b = 0.32$, $t(2216) = 3.13$, $p = .002$, $\beta = 0.220$, 95% CI [0.082, 0.358]. At T30 we found full mediation, with inoculation no longer being significant directly, $b = 0.05$, $t(2216) = 0.526$, $p = .599$, $\beta = 0.031$, 95% CI [-0.085, 0.148], but memory, $b = 0.17$, $t(2216) = 11.58$, $p < .001$, $\beta = 0.249$, 95% CI [0.215, 0.303], and motivation, $b = 0.16$, $t(2216) = 7.77$, $p < .001$, $\beta = 0.158$, 95% CI [0.118, 0.198], having a remaining influence, whilst inoculation directly influenced memory, $b = 2.50$, $t(2217) = 22.11$, $p < .001$, $\beta = 1.133$, 95% CI [1.032, 1.233], and motivation, $b = 0.28$, $t(2217) = 3.44$, $p < .001$, $\beta = 0.194$, 95% CI [0.084, 0.305]. See Supplementary Table 9 for the complete model estimates.

The data in this study allowed us to go one step further in our SEM analyses than Study 1 and Study 2 allowed, as due to the immediate posttest and a second posttest at a later date, we now have longitudinal data for the mapping of paths between time points. To test the memory-motivation model in its entirety, we therefore created an SEM model that includes inoculation at T0, memory at T0 and T30, motivation at T0 and T30, and the booster interventions at T10. See Fig. 6 for a simplified visual representation of the memory-motivation SEM model in Study 5. As can be seen from the estimates provided in the estimates table (see Supplementary Table 9) and the visual summary (Fig. 6), the video inoculation effects work indirectly via memory and motivation, with the largest effects for memory, both for inoculation on memory, and for memory on manipulativeness discernment performance. The role of motivation seems to be particularly important for the T0 memory formation and relatedly, the motivation booster presented at T10 did not provide any

additional benefits for performance or motivation at T30. Meanwhile, the memory booster presented at T10 successfully managed to boost the inoculation effect at T30 by boosting the inoculation memory, which in turn was the best predictor for the effect retention at T30. These findings are in line with the memory-motivation model of inoculation.

Supplementary Note 8: *(Study 5) Underlying Mechanisms Dominance Analysis*

We performed a dominance analysis to investigate the most dominant predictors of the inoculation effect at T30, and found that memory (41% dominance) and motivational threat (27%) were the best predictors of inoculation longevity. For a complete overview, see Supplementary Table 10.

To further demonstrate the role of memory in inoculation, we looked at the effect of the inoculation intervention for people who have a good memory of the intervention at T30, and found a large effect, $M_{\text{diff}} = 1.01$, $t(896) = 10.76$, $p_{\text{tukey}} < .001$, $d = 0.728$, 95% CI [0.591, 0.865], for manipulativeness at 29 days, while only a small effect was found for those with an average memory of the intervention, $M_{\text{diff}} = 0.31$, $t(1471) = 3.68$, $p_{\text{tukey}} < .001$, $d = 0.220$, 95% CI [0.102, 0.337].

Supplementary Note 9: *(Study 5) Open Memory Questions Word Cloud Analysis*

As a final exploratory analysis we used a concept mapping question as a qualitative analysis of participants' memory recall in Study 5. Participants were asked to write down concepts they remembered from the original video in an open box, before they received directed memory questions. We used natural language processing packages in R—SnowballC [53], tm [54], and wordcloud [1]—to clean the text data and mapped the data by counting the frequency of the words entered. The results of this question—which can be seen in Supplementary Fig. 8—show that the inoculated groups have a distinct memory network at T30, that even before prompting participants with direct memory questions, they were able to recall key concepts of the inoculation intervention (e.g., "emotional", "manipulative", "language"). The control group participants recalled concepts from the control video (e.g., "eye", "macular", "degeneration").

References

1. Fellows, I. wordcloud: Word Clouds. *Comprehensive R Archive Network* https://cran.r-project.org/package=wordcloud (2018).
2. Neuwirth, E. RColorBrewer: ColorBrewer Palettes. *Comprehensive R Archive Network* https://cran.r-project.org/package=RColorBrewer (2022).
3. van der Linden, S., Leiserowitz, A., Rosenthal, S. & Maibach, E. Inoculating the public against misinformation about climate change. *Global Chall.* **1**, 1600008 (2017).
4. Maertens, R., Anseel, F. & van der Linden, S. Combatting climate change misinformation: Evidence for longevity of inoculation and consensus messaging effects. *J. Environ. Psychol.* **70**, Article 101455 (2020).
5. Cook, J. *et al.* Consensus on consensus: A synthesis of consensus estimates on human-caused global warming. *Environ. Res. Lett.* **11**, 048002 (2016).
6. van der Linden, S., Leiserowitz, A., Feinberg, G. D. & Maibach, E. How to communicate the scientific consensus on climate change: Plain facts, pie charts or metaphors? *Clim. Change* **126**, 255–262 (2014).
7. Readfearn, G. Revealed: Most popular climate story on social media told half a million people the science was a hoax. https://www.desmogblog.com/2016/11/29/revealed-most-popular-climate-story-social-media-told-half-million-people-science-was-hoax (2016).
8. Roozenbeek, J. & van der Linden, S. Fake news game confers psychological resistance against online misinformation. *Palgrave Communications* **5**, 1–10 (2019).
9. Maertens, R., Roozenbeek, J., Basol, M. & van der Linden, S. Long-term effectiveness of inoculation against misinformation: Three longitudinal experiments. *J. Exp. Psychol. Appl.* (2021) doi:10.1037/xap0000315.

10. Roozenbeek, J., van der Linden, S., Goldberg, B., Rathje, S. & Lewandowsky, S. Psychological inoculation improves resilience against misinformation on social media. *Sci Adv* **8**, eabo6254 (2022).
11. Lewandowsky, S., Oreskes, N., Risbey, J. S., Newell, B. R. & Smithson, M. Seepage: Climate change denial and its effect on the scientific community. *Glob. Environ. Change* **33**, 1–13 (2015).
12. Lewandowsky, S., Pilditch, T. D., Madsen, J. K., Oreskes, N. & Risbey, J. S. Influence and seepage: An evidence-resistant minority can affect public opinion and scientific belief formation. *Cognition* **188**, 124–139 (2019).
13. van der Linden, S., Leiserowitz, A., Feinberg, G. D. & Maibach, E. The scientific consensus on climate change as a gateway belief: Experimental evidence. *PLoS One* **10**, e0118489 (2015).
14. van der Linden, S., Leiserowitz, A. & Maibach, E. The gateway belief model: A large-scale replication. *Journal of Environmental Psychology* **62**, 49–58 (2019).
15. Cook, J., Lewandowsky, S. & Ecker, U. K. H. Neutralizing misinformation through inoculation: Exposing misleading argumentation techniques reduces their influence. *PLoS One* **12**, Article e0175799 (2017).
16. van der Linden, S. & Roozenbeek, J. Psychological inoculation against fake news. in *The psychology of fake news: Accepting, sharing, and correcting misinformation* (eds. Greifeneder, R., Jaffé, M., Newman, E. J. & Schwarz, N.) (Psychology Press, 2020).
17. Traberg, C. S., Roozenbeek, J. & van der Linden, S. Psychological Inoculation against Misinformation: Current Evidence and Future Directions. *Ann. Am. Acad. Pol. Soc. Sci.* **700**, 136–151 (2022).

18. Roozenbeek, J., van der Linden, S. & Nygren, T. Prebunking interventions based on the psychological theory of ``inoculation'' can reduce susceptibility to misinformation across cultures. *Harvard Kennedy School Misinformation Review* **1**, 1–23 (2020).

19. Basol, M., Roozenbeek, J. & van der Linden, S. Good news about Bad News: Gamified inoculation boosts confidence and cognitive immunity against fake news. *J Cogn* **3**, 1–9 (2020).

20. Banas, J. A. & Rains, S. A. A meta-analysis of research on inoculation theory. *Commun. Monogr.* **77**, 281–311 (2010).

21. Ivanov, B., Parker, K. A. & Dillingham, L. L. Testing the limits of inoculation-generated resistance. *West. J. Speech Commun.* **82**, 648–665 (2018).

22. Compton, J. Threat and/in Inoculation Theory. *Int. J. Commun. Syst.* **15**, 13 (2021).

23. Compton, J., Ivanov, B. & Hester, E. Inoculation Theory and Affect. *Int. J. Commun. Syst.* **16**, 14 (2022).

24. Compton, J. Inoculation theory. in *The Sage handbook of persuasion: Developments in theory and practice* (eds. Dillard, J. P. & Shen, L.) 220–237 (Sage, Thousand Oaks, CA, 2013).

25. McGuire, W. J. The effectiveness of supportive and refutational defenses in immunizing and restoring beliefs against persuasion. *Sociometry* **24**, 184–197 (1961).

26. McGuire, W. J. Resistance to persuasion conferred by active and passive prior refutation of the same and alternative counterarguments. *J. Abnorm. Soc. Psychol.* **63**, 326 (1961).

27. McGuire, W. J. Persistence of the resistance to persuasion induced by various types of prior belief defenses. *The Journal of Abnormal and Social Psychology* **64**, 241–248 (1962).

28. McGuire, W. J. Inducing resistance to persuasion: Some contemporary approaches. *Adv. Exp. Soc. Psychol.* **1**, 191–229 (1964).

29. McGuire, W. J. Persuasion, resistance, and attitude change. *Handbook of communication* **1**, (1973).

30. McGuire, W. J. & Papageorgis, D. The relative efficacy of various types of prior belief-defense in producing immunity against persuasion. *J. Abnorm. Soc. Psychol.* **62**, 327–337 (1961).

31. McGuire, W. J. & Papageorgis, D. Effectiveness of forewarning in developing resistance to persuasion. *Public Opin. Q.* **26**, 24–34 (1962).

32. Banas, J. A. & Richards, A. S. Apprehension or motivation to defend attitudes? Exploring the underlying threat mechanism in inoculation-induced resistance to persuasion. *null* **84**, 164–178 (2017).

33. Richards, A. S. & Banas, J. A. The opposing mediational effects of apprehensive threat and motivational threat when inoculating against reactance to health promotion. *Southern Communication Journal* **83**, 245–255 (2018).

34. Ivanov, B. Designing inoculation messages for health communication campaigns. in *Health communication message design: Theory and practice* (ed. Cho, H.) 73–93 (Sage, Thousand Oaks, CA, 2012).

35. Pfau, M. *et al.* Inoculation and mental processing: The instrumental role of associative networks in the process of resistance to counterattitudinal influence. *Commun. Monogr.* **72**, 414–441 (2005).

36. Collins, A. M. & Loftus, E. F. A spreading-activation theory of semantic processing. *Psychol. Rev.* **82**, 407 (1975).

37. Ebbinghaus, H. *Über Das Gedächtnis: Untersuchungen Zur Experimentellen Psychologie*. (Duncker & Humblot, 1885).

38. Frankland, P. W. & Bontempi, B. The organization of recent and remote memories. *Nat. Rev. Neurosci.* **6**, 119–130 (2005).

39. Hardt, O., Nader, K. & Nadel, L. Decay happens: The role of active forgetting in memory. *Trends Cogn. Sci.* **17**, 111–120 (2013).

40. Murre, J. M. J. & Dros, J. Replication and analysis of Ebbinghaus' forgetting curve. *PLoS One* **10**, e0120644 (2015).

41. Murty, V. P. & Dickerson, K. C. Motivational Influences on Memory. in *Recent Developments in Neuroscience Research on Human Motivation* vol. 19 203–227 (Emerald Group Publishing Limited, 2016).

42. Smith, E. R. Mental representation and memory. *The handbook of social psychology* **2**, 391–445 (1998).

43. Hill, S. J., Lo, J., Vavreck, L. & Zaller, J. How quickly we forget: The duration of persuasion effects from mass communication. *Political Communication* **30**, 521–547 (2013).

44. Lecheler, S. & de Vreese, C. H. How Long Do News Framing Effects Last? A Systematic review of Longitudinal Studies. *Annals of the International Communication Association* **40**, 3–30 (2016).

45. Nisa, C. F., Bélanger, J. J., Schumpe, B. M. & Faller, D. G. Meta-analysis of randomised controlled trials testing behavioural interventions to promote household action on climate change. *Nat. Commun.* **10**, Article 4545 (2019).

46. Roozenbeek, J., Maertens, R., McClanahan, W. & van der Linden, S. Disentangling Item and Testing Effects in Inoculation Research on Online Misinformation: Solomon Revisited. *Educ. Psychol. Meas.* **81**, 340–362 (2021).

47. Karpicke, J. D. & Roediger, H. L., 3rd. The critical importance of retrieval for learning. *Science* **319**, 966–968 (2008).

48. Linton, M. Memory for real world events. in *Explorations in cognition* (eds. Norman, D. A. & Rumelhart, D. E.) 376–404 (W.H. Freeman, New York, NY, 1975).

49. Roediger, H. L. & Karpicke, J. D. Test-enhanced learning: taking memory tests improves long-term retention. *Psychol. Sci.* **17**, 249–255 (2006).

50. Roediger, H. L., 3rd & Karpicke, J. D. The power of testing memory: Basic research and implications for educational practice. *Perspect. Psychol. Sci.* **1**, 181–210 (2006).

51. Budescu, D. V. Dominance analysis: A new approach to the problem of relative importance of predictors in multiple regression. *Psychol. Bull.* **114**, 542–551 (1993).

52. Rosseel, Y. Lavaan: An R package for structural equation modeling and more. *J. Stat. Softw.* **48**, 1–36 (2012).

53. Bouchet-Valat, M. SnowballC: Snowball stemmers based on the C 'libstemmer' UTF-8 library. *Comprehensive R Archive Network* https://cran.r-project.org/web/packages/SnowballC (2020).

54. Theußl, S., Feinerer, I. & Hornik, K. A tm plug-in for distributed text mining in R. *J. Stat. Softw.* **51**, 1–31 (2012).
