# Supplementary material for: Psychological booster shots targeting memory increase long-term resistance against misinformation
Source: Nat Commun. 2025 Mar 11;16:2062. doi: 10.1038/s41467-025-57205-x (PMC11897321; doi:10.1038/s41467-025-57205-x)
Supplement: Supplementary file 2 — Reporting Summary [file 41467_2025_57205_MOESM2_ESM.pdf]

nature portfolio

Corresponding author(s): Dr Rakoen Maertens

Last updated by author(s): Sep 20, 2024

Reporting Summary

Nature Portfolio wishes to improve the reproducibility of the work that we publish. This form provides structure for consistency and transparency in reporting. For further information on Nature Portfolio policies, see our Editorial Policies and the Editorial Policy Checklist.

Statistics

For all statistical analyses, confirm that the following items are present in the figure legend, table legend, main text, or Methods section.

n/a	Confirmed	
☐	☒	The exact sample size (n) for each experimental group/condition, given as a discrete number and unit of measurement
☐	☒	A statement on whether measurements were taken from distinct samples or whether the same sample was measured repeatedly
☐	☒	The statistical test(s) used AND whether they are one- or two-sided<br>*Only common tests should be described solely by name; describe more complex techniques in the Methods section.*
☐	☒	A description of all covariates tested
☐	☒	A description of any assumptions or corrections, such as tests of normality and adjustment for multiple comparisons
☐	☒	A full description of the statistical parameters including central tendency (e.g. means) or other basic estimates (e.g. regression coefficient) AND variation (e.g. standard deviation) or associated estimates of uncertainty (e.g. confidence intervals)
☐	☒	For null hypothesis testing, the test statistic (e.g. F, t, r) with confidence intervals, effect sizes, degrees of freedom and P value noted<br>*Give P values as exact values whenever suitable.*
☐	☒	For Bayesian analysis, information on the choice of priors and Markov chain Monte Carlo settings
☐	☒	For hierarchical and complex designs, identification of the appropriate level for tests and full reporting of outcomes
☐	☒	Estimates of effect sizes (e.g. Cohen's d, Pearson's r), indicating how they were calculated

Our web collection on statistics for biologists contains articles on many of the points above.

Software and code

Policy information about availability of computer code

Data collection	Qualtrics (v2021, v2022) was used for the survey experiment set up and data collection.
Data analysis	The Qualtrics survey configuration files, R cleaning scripts, and R analysis scripts for this work have been deposited in the Open Science Framework (OSF) repositories for each study, where they are publicly accessible at https://doi.org/10.17605/OSF.IO/9ZXJE (Study 1), https://doi.org/10.17605/OSF.IO/HWMGE (Study 2), and https://doi.org/10.17605/OSF.IO/ZRQ87 (Studies 3–5). All data analyses were done in R (4.0, 4.1, .4.2, 4.3, 4.4), with the following additional packages: BayesFactor (0.9.12.4.7), car (3.1.2), cowplot (1.1.3), domir (1.2.0), dplyr (1.1.4), effectsize (0.8.9), ggplot2 (3.5.1), jamm (1.2.4), jmv (2.5.6), jmvcore (2.6.3), lavaan (0.6.18), NLP (0.3.0), openxlsx (4.2.7), plyr (1.8.9), psych (2.4.6.26), purrr (1.0.2), RColorBrewer (1.1.3), reshape2 (1.4.4), Rmisc (1.5.1), scatr (1.0.1), semPlot (1.1.6), semTools (0.5.6), SnowballC (0.7.1), stats (4.4.0), tibble (3.2.1), tm (0.7.14), wordcloud (2.6), writexl (1.5.0).

For manuscripts utilizing custom algorithms or software that are central to the research but not yet described in published literature, software must be made available to editors and reviewers. We strongly encourage code deposition in a community repository (e.g. GitHub). See the Nature Portfolio guidelines for submitting code & software for further information.

Data

Policy information about availability of data

All manuscripts must include a data availability statement. This statement should provide the following information, where applicable:

- Accession codes, unique identifiers, or web links for publicly available datasets
- A description of any restrictions on data availability
- For clinical datasets or third party data, please ensure that the statement adheres to our policy

Supplementary Information is available for this paper. The supplements, raw datasets, clean datasets, survey printouts, preregistrations, and materials related to this work have been deposited in the Open Science Framework (OSF) repositories for each study, where they are publicly accessible at https://doi.org/10.17605/OSF.IO/9ZXJE (Study 1), https://doi.org/10.17605/OSF.IO/HWMGE (Study 2), and https://doi.org/10.17605/OSF.IO/ZRQ87 (Studies 3–5).

Research involving human participants, their data, or biological material

Policy information about studies with human participants or human data. See also policy information about sex, gender (identity/presentation), and sexual orientation and race, ethnicity and racism.

Reporting on sex and gender

We collected gender but not sex. For each study we also record self-identified gender to ensure representativeness and balance in the data, but additional gender analyses were not conducted as this was not a research question, not preregistered, and could lead to incorrect conclusions due to limited power. You can find the gender distribution listed here below:

*Study 1: 49.21% identified as male (48.22% as female; 2.03% as non-binary; 0.33% as transgender, 0.22% as “other”)

* Study 2: 54.30% identified as female (41.39% as male; 3.12% as non-binary; 1.04% as transgender, 0.15% as “other”)

* Study 3: 50.70% identified as female (48.26% as male; 0.86% as non-binary; 0.05% as “other”; 0.14% preferred not to answer), the average age was 46.00 (SD = 16.41, Mdn = 46)

* Study 4: 51.73% identified as female (47.65% as male; 0.44% as non-binary; 0.10% as “other”; 0.08% preferred not to answer)

* Study 5: 55.14% identified as female (44.50% as male; 0.23% as non-binary; 0.09% as “other”; 0.05% preferred not to answer)

Reporting on race, ethnicity, or other socially relevant groupings

Other than gender (see above), we also collected self-reported political party affiliation of participants for each study, to make sure that the major political parties in the US are represented, which is important as the topic of misinformation explored in this study includes what could be perceived as political political misinformation. An overview of this data for those who completed the entire study can be found here:

* Study 1: Of those participants who indicated their political party and completed the study, 894 identified as Democrats (55.1%), 538 as Independents (33.1%), and 191 as Republicans (11.8%).

* Study 2: Political party affiliation data was not available.

* Study 3: Of those participants who indicated their political party and completed the study, 837 identified as Democrats (38.6%), 657 as Independents (30.3%), and 672 as Republicans (31.0%).

* Study 4: Of those participants who indicated their political party and completed the study, 1,128 identified as Democrats (37.7%), 947 as Independents (31.7%), and 916 as Republicans (30.6%).

* Study 5: Of those participants who indicated their political party and completed the study, 802 identified as Democrats (37.2%), 641 as Independents (29.7%), and 712 as Republicans (33.0%).

Population characteristics

* Study 1: The average age was 35.79 (SD = 13.07, Mdn = 33), 58.69% had a higher education degree, 62.58% identified as left-wing (22.47% as centrist; 14.96% as right-wing), 65.59% used social media multiple times a day (19.29% once a day, 7.29% weekly, 4.99% less often than weekly, 2.85% never), and 22.19% used Twitter multiple times a day (13.86% once a day, 12.06% weekly, 19.07% less often than weekly, 32.82% never).

* Study 2: The average age was 33.18 (SD = 12.25, Mdn = 30), 53.12% had a higher education degree, 66.17% identified as left-wing (22.40% as centrist; 11.42% as right-wing), 68.55% used social media multiple times a day (17.66% once a day, 6.08% weekly, 4.75% less often than weekly, 2.97% never), and 24.63% used Twitter multiple times a day (15.88% once a day, 12.17% weekly, 21.66% less often than weekly, 25.67% never).

* Study 3: The average age was 46.00 (SD = 16.41, Mdn = 46), 66.70% had a higher education degree (1.85% did not finish high school), 31.73% identified as left-wing (32.85% as centrist; 35.42% as right-wing), 39.84% checked the news multiple times a day (34.48% once a day; 14.65% weekly; 8.43% less often than weekly; 2.25% never), 54.08% used social media multiple times a day (23.43% once a day; 9.55% weekly; 5.36% less often than weekly; 7.57% never), 29.11% used YouTube multiple times a day (22.89% once a day; 26.32% weekly; 10.69% less often than weekly; 5.59% never), and 6.17% used YouTube for news consumption multiple times a day (12.89% once a day; 16.54% weekly; 28.39% less often than weekly; 40.42% never).

* Study 4: The average age was 45.79 (SD = 16.46, Mdn = 45), 65.63% had a higher education degree (1.35% did not finish high school), 30.47% identified as left-wing (35.51% as centrist; 34.02% as right-wing), 36.57% checked the news multiple times a day (35.72% once a day; 14.87% weekly; 9.83% less often than weekly; 3.01% never), 51.05% used social media multiple times a day (25.16% once a day; 10.81% weekly; 6.16% less often than weekly; 6.82% never), 27.11% used YouTube multiple times a day (22.59% once a day; 27.84% weekly; 16.74% less often than weekly; 5.73% never), and 5.83% used YouTube for news consumption multiple times a day (11.28% once a day; 15.25% weekly; 22.13% less often than weekly; 45.51% never).

* Study 5: The average age was 53.29 (SD = 14.48, Mdn = 55), 67.48% had a higher education degree (1.40% did not complete high school), 29.19% identified as left-wing (34.23% as centrist; 36.58% as right-wing), 40.90% checked the news multiple times a day (36.85% once a day; 12.52% weekly; 7.70% less often than weekly; 2.03% never), 45.68% used social media multiple times a day (25.09% once a day; 10.90% weekly; 7.21% less often than weekly; 11.13% never), 21.89% used YouTube multiple times a day (19.77% once a day; 29.59% weekly; 21.08% less often than weekly; 7.66% never), and 5.72% used YouTube for news consumption multiple times a day (9.86% once a day; 11.49% weekly; 21.62% less often than weekly; 51.31% never).

Recruitment

The sample consisted of a general U.S. population, with participants of 18 years or older. For Studies 1 and 2 we recruited Prolific convenience samples with gender balancing. For Studies 3, 4, and 5 we recruited, using the Bilendi & respondi services, representative inter-locking hard quota samples to ensure demographic alignment with U.S. census data (2019) for age and gender. These samples were chosen to ensure comparability with prior research using these interventions. The sample sizes were determined based on expected effect sizes from previous studies with similar paradigms, targeting a power of 0.95 and an alpha level of 0.05. For study-specific information, see below:

* Study 1 and Study 2: We used convenience sampling of the general U.S. population through the Prolific platform, with a balanced gender ratio, and participants of 18 years or older only.
- Study 1: N = 2,657 (N = 1,825 after exclusions).
- Study 2: N = 1,350 (N = 674 after exclusions).

* Study 3, Study 4, and Study 5: We used representative quota sampling of the general U.S. population with the recruitment services provided by Respondi/Bilendi. We applied hard interlocking quotas for age and gender, based on the 2019 U.S. Census data. All participants were 18 years or older.
- Study 3: N = 2,895 (N = 2,219 after exclusions).
- Study 4: N = 5,191 (N = 4,821 after exclusions).
- Study 5: N = 6,164 (N = 2,220 after exclusions).

The studies did not have any specific requirements to be able to participate, and we revealed only a relatively neutral general theme at the recruitment and informed consent stages, mentioning that it was a study about news/media and misinformation, without elaborating on the subtopics. We therefore expect self-selection bias to be minimal on this basis.

Ethics oversight

The study protocols for Study 1 (ref. PRE.2021.086), Study 2 (ref. PRE.2021.087), and Studies 3–5 (ref. PRE.2021.012), were all reviewed and approved by the Cambridge Psychology Research Ethics Committee at the University of Cambridge prior to the start of data collection. Informed consent was obtained from the participants at the start of each study, and all data has been anonymized prior to publication. Participants in Studies 1–2 were compensated at ~£5.00 per hour, while for Studies 3–5 the market research company Bilendi & respondi was responsible for rewarding the participants. Participants completed the study via an online Qualtrics survey in a self-directed manner, ensuring they were not influenced by the researchers during data collection. The study goals were partly revealed at the informed consent stage of the study, but participants did not know all details nor the full purpose of the experimental conditions. The participants were in general blind to the exact experimental condition they were randomly allocated to. For each study we also record self-identified gender to ensure representativeness and balance in the data, but additional gender analyses were not conducted as this was not a research question, not preregistered, and could lead to incorrect conclusions due to limited power. We also collected self-reported political party affiliation of participants for each study, to make sure that the major political parties in the US are represented, which is important as the topic of misinformation explored in this study includes what could be perceived as political political misinformation. At the end of the study, the participants were debriefed and were given the opportunity to have their data removed, but no one asked for their data to be removed.

Note that full information on the approval of the study protocol must also be provided in the manuscript.

Field-specific reporting

Please select the one below that is the best fit for your research. If you are not sure, read the appropriate sections before making your selection.

☐ Life sciences ☒ Behavioural & social sciences ☐ Ecological, evolutionary & environmental sciences

For a reference copy of the document with all sections, see nature.com/documents/nr-reporting-summary-flat.pdf

Behavioural & social sciences study design

All studies must disclose on these points even when the disclosure is negative.

Study description

This work employed five quantitative, experimental, and longitudinal online survey designs to investigate the effects of inoculation interventions on resilience to misinformation, and the role of memory and motivation on the effectiveness over time. Participants were randomly allocated to one of several experimental conditions, including a control condition, an inoculation condition, and a

booster condition. The study measured participants' responses at multiple time points after the intervention (immediately after, ~10 days after, and ~30 days after) to assess their resilience to misinformation.

Research sample

The sample consisted of a general U.S. population, with participants of 18 years or older. For Studies 1 and 2 we recruited Prolific convenience samples with gender balancing. For Studies 3, 4, and 5 we recruited, using the Bilendi & respondi services, representative inter-locking hard quota samples to ensure demographic alignment with U.S. census data (2019) for age and gender. These samples were chosen to ensure comparability with prior research using these interventions.

Sampling strategy

The sample sizes were determined based on expected effect sizes from previous studies with similar paradigms, targeting a power of 0.95 and an alpha level of 0.05. For study-specific information, see below:

* Study 1 and Study 2: We used convenience sampling of the general U.S. population through the Prolific platform, with a balanced gender ratio, and participants of 18 years or older only.
- Study 1: N = 832 exclusions (N = 2,657 recruited, N = 1,825 after exclusions).
- Study 2: N = 676 exclusions (N = 1,350 recruited, N = 674 after exclusions).

* Study 3, Study 4, and Study 5: We used representative quota sampling of the general U.S. population with the recruitment services provided by Respondi/Bilendi. We applied hard interlocking quotas for age and gender, based on the 2019 U.S. Census data. All participants were 18 years or older.
- Study 3: N = 676 exclusions (N = 2,895 recruited, N = 2,219 after exclusions).
- Study 4: N = 370 exclusions (N = 5,191 recruited, N = 4,821 after exclusions).
- Study 5: N = 3,944 exclusions (N = 6,164 recruited, N = 2,220 after exclusions).

The studies did not have any specific requirements to be able to participate, and we revealed only a relatively neutral general theme at the recruitment and informed consent stages, mentioning that it was a study about news/media and misinformation, without elaborating on the subtopics. We therefore expect self-selection bias to be minimal on this basis.

Data collection

Participants completed the study via an online Qualtrics survey in a self-directed manner, ensuring they were not influenced by the researchers during data collection. The study goals were partly revealed at the informed consent stage of the study, but participants did not know all details nor the full purpose of the experimental conditions. The participants were in general blind to the exact experimental condition they were randomly allocated to. At the end of the study, the participants were debriefed and were given the opportunity to have their data removed, but no one asked for their data to be removed.

Timing

* Study 1:
- T1: 11/01/2022-13/01/2022
- T2: 19/01/2022-25/01/2022
- T3: 08/02/2022-14/02/2022

* Study 2:
- T1: 13/01/2022-26/01/2022
- T2: 22/01/2022-06/02/2022
- T3: 11/02/2022-22/02/2022

* Study 3:
- T1: 18/06/2021-14/07/2021
- T2: 30/06/2021-09/08/2021

* Study 4:
- T1: 09/09/2021-24/09/2021
- T2: 11/09/2021-26/09/2021
- T3: 17/09/2021-02/10/2021
- T4: 07/10/2021-22/10/2021

* Study 5:
- T1: 19/11/2021-09/12/2021
- T2: 27/11/2021-18/12/2021
- T3: 15/12/2021-07/01/2022

Data exclusions

Exclusion criteria were made to ensure high-quality data and were preregistered. Exact number of exclusions can be found above. Exclusion criteria can be found below:

* Study 1: participants were excluded when they 1) failed the manipulation check, 2) failed both attention checks, 3) participated in the survey multiple times, or 4) did not complete the entire survey. We also excluded participants who did not participate within a window of 3 days from the intended participation date (i.e., 3 days before or after).

* Study 2: participants were excluded when they 1) failed the manipulation check, 2) failed both attention checks, 3) participated in the survey multiple times, or 4) did not complete the entire survey. We also excluded participants who did not participate in the follow-up within 3 days from the intended participation date.

* Study 3: removing participants who did not complete the entire study, failed both the manipulation and the attention check, participated multiple times, or entered the same response to each of the items of the dependent variable.

* Study 4: participants that failed both the manipulation check and attention check, participated in the survey more than once, entered the same response to all items of the dependent variable, or did not complete the entire survey.

	* Study 5: we excluded incomplete and low-quality responses, participants that did not participate in all three parts of the survey, and participants who did not participate in the follow-up sessions within 3 days before or after the intended time (T10: 10 days after, T30: 30 days after).
Non-participation	Across all five studies, participants (N = 25,885) were presented with informed consent before proceeding with the survey. 2,070 (8.0%) participants declined to provide informed consent and therefore did not start the study. In Study 1, the response rate was 99.9%, with 2,897 participants consenting out of 2,901 approached. In Study 2, the response rate was 99.7%, with 1,580 participants consenting out of 1,585. For Study 3, the response rate was 91.9%, with 5,333 participants consenting out of 5,804 approached. In Study 4, the response rate was 91.5%, with 6,080 participants consenting out of 6,643 approached. Finally, in Study 5, the response rate was 88.5%, with 7,925 participants consenting out of 8,952 approached. Reasons for declining were not recorded.<br><br>Of those who provided consent, 2,816 (11.8%) dropped out before the end of the first part of the study (i.e., before completing timepoint 1). In Study 1, the dropout rate was 3.0%, with 85 participants dropping out out of 2,897 who consented. In Study 2, the dropout rate was 14.0%, with 221 participants dropping out out of 1,580 who consented. For Study 3, the dropout rate was 6.8%, with 365 participants dropping out out of 5,333 who consented. In Study 4, the dropout rate was 13.1%, with 799 participants dropping out out of 6,080 who consented. Finally, in Study 5, the dropout rate was 17.0%, with 1,346 participants dropping out out of 7,925 who consented. Reasons for dropping out were not recorded.
Randomization	Random allocation.

Reporting for specific materials, systems and methods

We require information from authors about some types of materials, experimental systems and methods used in many studies. Here, indicate whether each material, system or method listed is relevant to your study. If you are not sure if a list item applies to your research, read the appropriate section before selecting a response.

Materials & experimental systems

n/a	Involved in the study
☒	☐ Antibodies
☒	☐ Eukaryotic cell lines
☒	☐ Palaeontology and archaeology
☒	☐ Animals and other organisms
☒	☐ Clinical data
☒	☐ Dual use research of concern
☒	☐ Plants

Methods

n/a	Involved in the study
☒	☐ ChIP-seq
☒	☐ Flow cytometry
☒	☐ MRI-based neuroimaging

Plants

Seed stocks	*Report on the source of all seed stocks or other plant material used. If applicable, state the seed stock centre and catalogue number. If plant specimens were collected from the field, describe the collection location, date and sampling procedures.*
Novel plant genotypes	*Describe the methods by which all novel plant genotypes were produced. This includes those generated by transgenic approaches, gene editing, chemical/radiation-based mutagenesis and hybridization. For transgenic lines, describe the transformation method, the number of independent lines analyzed and the generation upon which experiments were performed. For gene-edited lines, describe the editor used, the endogenous sequence targeted for editing, the targeting guide RNA sequence (if applicable) and how the editor was applied.*
Authentication	*Describe any authentication procedures for each seed stock used or novel genotype generated. Describe any experiments used to assess the effect of a mutation and, where applicable, how potential secondary effects (e.g. second site T-DNA insertions, mosiacism, off-target gene editing) were examined.*
